# Sputum and blood transcriptomics characterisation of the inhaled PDE4 inhibitor CHF6001 on top of triple therapy in patients with chronic bronchitis

**DOI:** 10.1186/s12931-020-1329-y

**Published:** 2020-03-20

**Authors:** Mirco Govoni, Michele Bassi, Stefano Vezzoli, Germano Lucci, Aida Emirova, Marie Anna Nandeuil, Stefano Petruzzelli, Gera L. Jellema, Ebenezer K. Afolabi, Brendan Colgan, Brian Leaker, Oliver Kornmann, Kai Michael Beeh, Henrik Watz, Dave Singh

**Affiliations:** 1 Global Clinical Development, Personalised Medicine and Biomarkers, Chiesi, Parma, Italy; 2 Almac Diagnostics, Craigavon, UK; 3 Celerion, Belfast, UK; 4 The Heart Lung Centre, London, UK; 5 IKF Pneumologie Frankfurt, Clinical Research Centre Respiratory Diseases, Frankfurt, Germany; 6 Insaf Respiratory Research Institute, Wiesbaden, Germany; 7 Pulmonary Research Institute at Lung Clinic Grosshansdorf, Airway Research Center North (ARCN), Member of the German Center for Lung Research (DZL), Grosshansdorf, Germany; 8 Medicines Evaluation Unit, The University of Manchester, Manchester University NHS Foundation Trust, Manchester, UK

**Keywords:** Gene expression, Inflammation, Phosphodiesterase 4 inhibitors, Biomarkers, Chronic obstructive pulmonary disease

## Abstract

**Background:**

Although phosphodiesterase-4 (PDE4) inhibitors have been shown to reduce COPD exacerbation rate, their biological mechanism of action is not completely elucidated at the molecular level. We aimed to characterise the whole genome gene expression profile of the inhaled PDE4-inhibitor CHF6001 on top of triple therapy in sputum cells and whole blood of patients with COPD and chronic bronchitis.

**Methods:**

Whole genome gene expression analysis was carried out by microarray in 54 patients before and after 32 days treatment with CHF6001 800 and 1600 μg and placebo twice daily (BID) in a randomised crossover study.

**Results:**

CHF6001 had a strong effect in sputum, with 1471 and 2598 significantly differentially-expressed probe-sets relative to placebo (p-adjusted for False Discovery Rate < 0.05) with 800 and 1600 μg BID, respectively. Functional enrichment analysis showed significant modulation of key inflammatory pathways involved in cytokine activity, pathogen-associated-pattern-recognition activity, oxidative stress and vitamin D with associated inhibition of downstream inflammatory effectors. A large number of pro-inflammatory genes coding for cytokines and matrix-metalloproteinases were significantly differentially expressed for both doses; the majority (> 87%) were downregulated, including macrophage inflammatory protein-1-alpha and 1-beta, interleukin-27-beta, interleukin-12-beta, interleukin-32, tumour necrosis factor-alpha-induced-protein-8, ligand-superfamily-member-15, and matrix-metalloproteinases-7,12 and 14. The effect in blood was not significant.

**Conclusions:**

Inhaled PDE4 inhibition by CHF6001 on top of triple therapy in patients with COPD and chronic bronchitis significantly modulated key inflammatory targets and pathways in the lung but not in blood. Mechanistically these findings support a targeted effect in the lung while minimising unwanted systemic class-effects.

**Trial registration:**

ClinicalTrial.gov, EudraCT, 2015–005550-35. Registered 15 July 2016.

## Background

The orally administered phosphodiesterase 4 (PDE4) inhibitor roflumilast has been shown to reduce the rate of exacerbations in patients with COPD who have a chronic bronchitis phenotype [1]. Clinical trials have demonstrated the greatest benefit is in patients who are at high risk of frequent or severe exacerbations despite inhaled corticosteroid (ICS) and long-acting β_2_ agonist therapy (LABA), with or without a long-acting muscarinic antagonist (LAMA) [2, 3]. However, systemic exposure after oral administration of roflumilast can cause side effects such as nausea, weight loss and gastrointestinal disturbance [4]. Furthermore, although in clinical practice a PDE4 inhibitor is often administered in addition to inhaled triple ICS/LABA/LAMA therapy [5], there is a lack of information regarding the anti-inflammatory effects of a PDE4 inhibitor administered in addition to maintenance triple therapy.

CHF6001 is a novel PDE4 inhibitor currently in clinical development that has been specifically formulated as an inhaled extrafine formulation (i.e., mass median aerodynamic diameter ≤ 2 μm) [6], and designed to have high protein binding and rapid elimination from the systemic circulation [7] thus maximising exposure in the lung and avoiding systemic adverse effects [8]. In a 24-week dose finding study, when administered in addition to LABA therapy, CHF6001 numerically reduced the exacerbation rate in patients with chronic bronchitis, with all doses being well tolerated [9].

We previously reported that CHF6001 significantly decreased the levels of various inflammatory mediators in the sputum of patients with COPD and a chronic bronchitis phenotype who are receiving inhaled maintenance triple therapy [10]. The current analyses aimed to validate and further characterise the biological effect of CHF6001 using high throughput gene expression analysis of the whole protein coding genome in sputum cells and blood in the patients recruited into the inflammatory mediator study [10].

## Methods

### Study objective and design

Samples were collected from a multicentre, three-way, placebo-controlled, double-blind crossover study, the results for which have been previously reported [10]. This study aimed to evaluate the effect of CHF6001 on inflammatory biomarkers in induced sputum and blood. The gene expression analyses described in this manuscript were a prespecified exploratory objective of the study.

After randomisation, eligible patients commenced three, 32-day treatment periods during which they received CHF6001 800 or 1600 μg twice daily (BID, total daily doses of 1600 or 3200 μg) or matching placebo, all via multi-dose dry-powder inhaler (NEXThaler). Treatment periods were separated by a 28–42 day washout. Induced sputum was collected pre-dose on Day 1, and 2 h post-dose on Days 20, 26 and 32 of each period, with blood samples collected pre-dose on Day 1 and 2 h post-dose on Day 32 (see the supplement for further detail, including methods for the sputum and blood collection, and processing for the ribonucleic acid [RNA] assessments, extraction and amplification, sample profiling, microarray data quality control and pre-processing). The trial was approved by the independent ethics committees at each institution, and was performed in accordance with the principles of the Declaration of Helsinki, and the International Conference on Harmonisation notes for guidance on Good Clinical Practice (ICH/CPMP/135/95). The trial is registered on ClinicalTrials.gov (NCT03004417) and EudraCT (2015–005550-35#). There were no protocol amendments. The data discussed in this publication have been deposited in NCBI’s Gene Expression Omnibus and are accessible through GEO Series accession number GSE133513 (https://www.ncbi.nlm.nih.gov/geo/query/acc.cgi?acc=GSE133513).

### Patients

Eligible patients were male or female, ≥40 years of age, current or ex-smokers with a smoking history ≥10 pack-years, a diagnosis of COPD, post-bronchodilator forced expiratory volume in 1 s (FEV_1_) ≥ 30 and < 70% predicted, ratio of FEV_1_ to forced vital capacity < 0.70, COPD Assessment Test score ≥ 10, a history of chronic bronchitis (defined as chronic cough and sputum production for more than 3 months per year for at least two consecutive years), and treated with inhaled triple ICS/LABA/LAMA therapy for at least 2 months prior to enrolment. All patients provided written informed consent prior to any study-related procedure.

The key exclusion criteria were a moderate or severe COPD exacerbation within 6 weeks prior to entry or between screening and randomisation, and the use of PDE4 inhibitors within 2 months prior to entry. Full inclusion and exclusion criteria can be found in the supplement.

### Statistical analysis

For the sputum analyses, patients had to have a minimum of one sample on Day 20, 26 or 32 that passed quality control, with the latest sample used for the analyses. If no matching pre-dose sample was available, the pre-dose sample from the closest available period was used.

Any probe sets with statistically significantly different pre-dose expression between treatments were identified and excluded (see ANOVA analysis, supplementary methods). An ANCOVA model was fitted to identify probe sets responding differentially to treatment, with change from pre-dose to post-dose expression as dependent variable, and subject, period, treatment and pre-dose expression as independent variables. For all analyses, statistical significance was considered as a *p*-value adjusted for False Discovery Rate using the Benjamini-Hochberg method (pFDR) below 0.05 [11].

### Functional enrichment analysis

Differentially expressed probe set lists were annotated with gene identifiers using the latest annotation provided by Affymetrix for the Plus 2.0 array. Differential gene lists smaller than 10 genes were not used for functional enrichment; lists larger than 1000 genes were filtered based on fold change (FC) using a cut-off of 1.3. For multiple probe sets associated with one gene, the probe set with the lowest pFDR value was used for analysis. Using Gene Ontology (GO) annotations and commercial software Ingenuity® Pathway Analysis (IPA®, QIAGEN Inc., https://www.qiagenbioinformatics.com/products/ingenuity-pathway-analysis), functional enrichment analysis was conducted to identify and rank biological entities associated with gene sets of interest. Entities were ranked according to a statistically derived enrichment score and were adjusted for multiple testing (pFDR < 0.05). Simulation of perturbations for significant IPA® canonical pathways was carried out using Molecule Activity Predictor (MAP) to simulate the downstream consequences of up or downregulation of molecules mediated by the treatment.

### Gene interaction network analysis

The genes of interest were input into the STRING database (Search Tool for Retrieval of Interacting Genes/proteins) version 11.0 (https://string-db.org/) [12]. When multiple probe sets were associated with the same gene of interest, the probe set with the lowest pFDR value was considered. For probe sets associated with more than one gene, all genes were considered for the network analysis.

## Results

### Patients and samples

Fifty-nine patients gave consent for gene expression investigations and were randomised, 54 of whom completed the study and were eligible for analysis, having pre- and post-dose samples available for at least one active dose and placebo in blood and/or sputum (Table 1). Sputum weight and the number of sputum cells/g were similar for the two CHF6001 doses (Fig. S1). A small number of post-dose samples did not have matching pre-dose samples. An analysis of principal components on pre-dose samples showed no association between period (1, 2 or 3) and gene expression data (principal component [PC] 1, *p* = 0.71 in sputum, *p* = 0.48 in blood), or between treatment and gene expression data (PC1, *p* = 0.94 in sputum, *p* = 0.38 in blood) (Fig. S2). Missing pre-dose samples were therefore replaced with the pre-dose samples from the closest available period (see supplement). Following quality and RNA quantity assessment, 50 patients had pre- and post-dose blood samples available for analysis with both CHF6001 doses and placebo, 37 of whom had pre- and post-dose sputum samples available for analysis with CHF6001 800 μg BID and placebo, with 41 having samples available with CHF6001 1600 μg BID and placebo (see supplement).

**Table 1:** Baseline demographics and disease characteristics

Parameter	Analysed patients (*N* = 54)
Age (years), mean (SD)	65.6 (5.9)
Male gender, n (%)	38 (70)
Race, n (%)	
Caucasian	53 (98)
Asian	1 (2)
BMI (kg/m^2^), mean (SD)	26.2 (4.5)
Time since first COPD diagnosis (years), mean (SD)	9.3 (4.7)
Smoking status at screening, n (%)	
Ex-smoker	24 (44)
Current smoker	30 (56)
Post-bronchodilator FEV_1_ (L), mean (SD)	1.46 (0.43)
Post-bronchodilator FEV_1_ (% predicted), mean (SD)	50.1 (11.7)
30 to < 50% predicted, n (%)	26 (48)
50 to 70% predicted, n (%)	28 (52)
COPD Assessment Test, mean (SD)	20.4 (6.0)
Sputum characteristics, mean (SD)	
Total cell count (× 10^6^/g)	4.6 (5.9)
Neutrophil %	82.4 (9.0)
Macrophage %	11.1 (7.5)
Eosinophil %	3.9 (4.4)
Lymphocyte %	0.2 (0.3)
Epithelial cells %	2.5 (3.7)

Abbreviations: *BMI*, Body mass index; *COPD*, Chronic obstructive pulmonary disease; *FEV*_*1*_, Forced expiratory volume in 1 s

### Differential expression analysis in response to treatment

Filtering to remove any uninformative transcripts (see supplement) resulted in 45,163 and 44,355 reliably detected probe sets in blood and sputum, respectively, being available for analysis, corresponding to nearly the whole protein coding genome of 19,000 genes [13]. Notably, no probe sets had a pre-dose expression for either CHF6001 dose that significantly varied from the placebo pre-dose value.

**Fig. 1:**
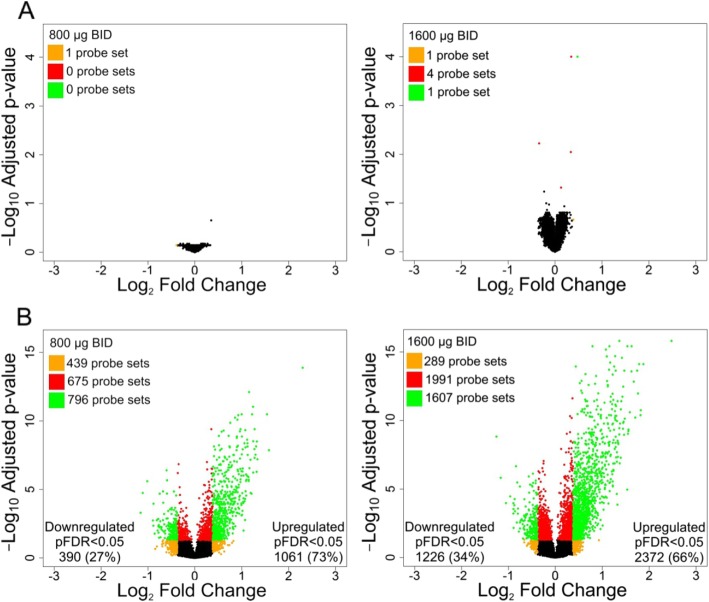
Differentially expressed probe sets for CHF6001 800 μg and 1600 μg BID relative to placebo in (A) blood and (B) sputum. Volcano plot depicting all detected probe sets and coloured by fold change (FC) and adjusted *p*-values: orange, |FC| > 1.3; red, pFDR< 0.05; green, |FC| > 1.3 and pFDR< 0.05. Abbreviation: BID, twice daily

The impact of treatment on gene expression in blood was minimal with either dose (Fig. 1a). In contrast, in sputum a large number of probe sets were differentially expressed between CHF6001 and placebo, the 1600 μg BID dose having the greatest effect (Fig. 1b). With CHF6001 800 μg BID, 1471 probe sets were significantly differentially expressed vs. placebo (pFDR< 0.05), 390 (27%) of which were downregulated and 1081 (73%) upregulated. CHF6001 1600 μg BID led to 3598 significantly differentially expressed probe sets vs. placebo, with 1226 (34%) downregulated and 2372 (66%) upregulated. Further, of the 1471 probe sets significantly differentially expressed with CHF6001 800 μg BID, 1229 (84%) were also significantly differentially expressed with CHF6001 1600 μg BID.

**Fig. 2:**
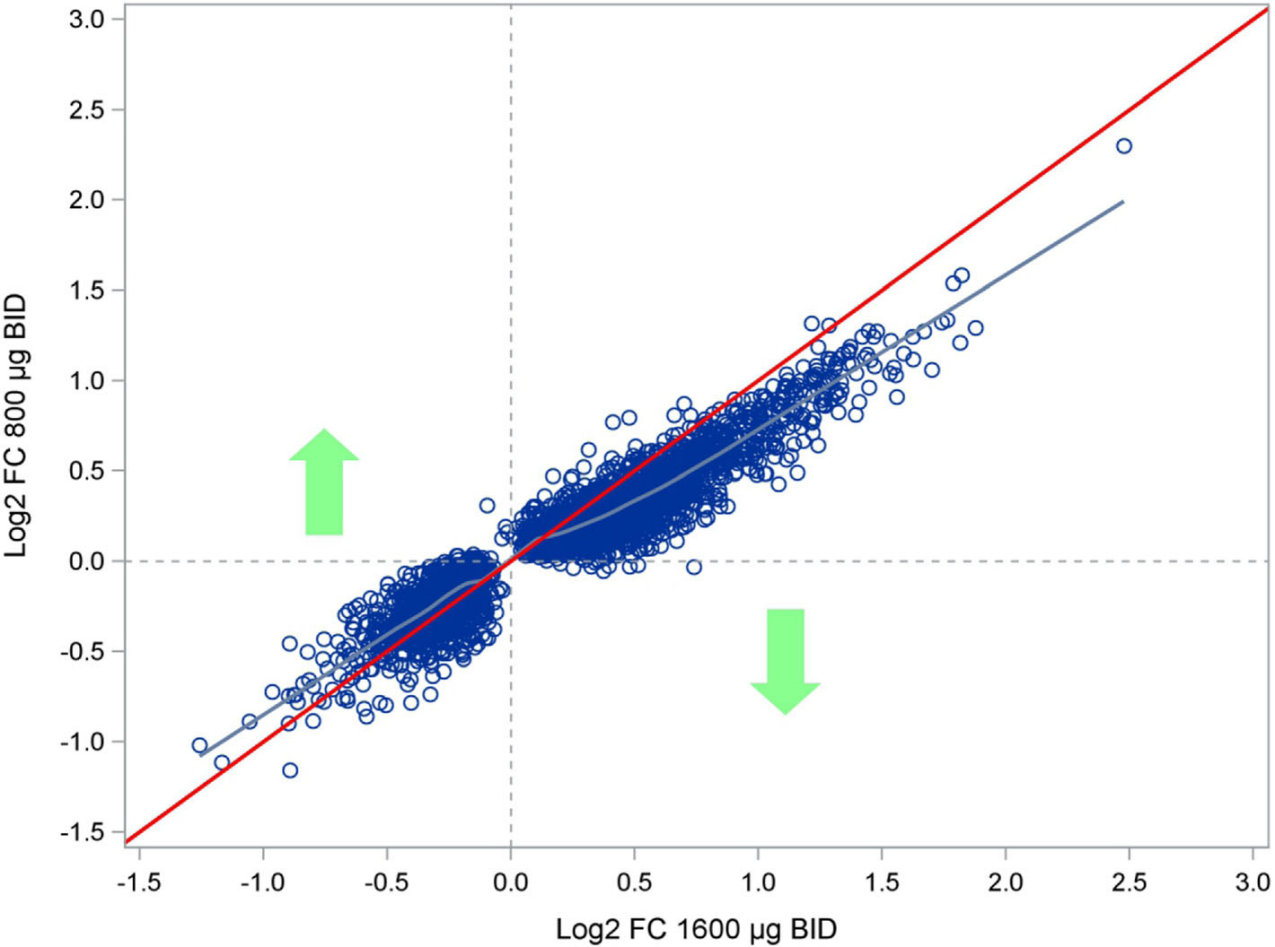
Log2 fold change with CHF6001 1600 μg BID vs. log2 fold change with CHF6001 800 μg BID. Equality line in red, fitted loess curve in blue. Only differentially expressed probe sets (pFDR< 0.05 for at least one dose) are presented. Abbreviation: BID, twice daily

Among the probe sets differentially expressed with at least one CHF6001 dose, > 99% followed the same direction for both treatments, i.e., either both up-regulated or both down-regulated (Fig. 2), with 90.1% of the upregulated probe sets and 79.1% of the downregulated probe sets showing a dose-response relationship.

### Functional enrichment analysis

In blood, functional enrichment analysis was not performed due to insufficient numbers of probe sets being available. In sputum, more than 1000 probe sets were significantly differentially expressed with at least one CHF6001 dose, so the analysis was restricted to probe sets with |FC| > 1.3. A total of 24 and 92 ingenuity canonical pathways were significantly modulated by CHF6001 800 and 1600 μg BID, respectively (Table S1), the most commonly associated terms being: immune response, cytokine signalling and inflammation, growth and development. Sixteen pathways associated with COPD pathophysiology were identified for CHF6001 800 μg BID, and 65 for CHF6001 1600 μg BID, covering cytokines, cyclic adenosine monophosphate (cAMP), T-helper (Th) 1 and 2 cells, dendritic cells, natural killer cells, oxidative stress, and vitamin D pathways (Table S1), with nine pathways in common between the two CHF6001 doses.

Simulation of perturbations for all significant IPA® canonical pathways was carried out for CHF6001 1600 μg BID using Molecule Activity Predictor to simulate the downstream consequences of treatment. This identified several key COPD pathways associated with consistent actual downregulation and/or predicted inhibition of downstream inflammatory mediators or mechanisms (Fig. S3) namely, *C-C chemokine receptor type 5 (CCR5) signalling in macrophages, chemokine signalling, high mobility group-B1 (HMGB1) signalling*, *triggering receptor expressed on myeloid cells 1 (TREM1) signalling*, *interleukin* (*IL)22 signalling, ceramide signalling, protein kinase c theta (PKC*θ*) signalling in T lymphocytes, role of pattern recognition receptors in recognition of bacteria and viruses*, *toll-like receptors signalling, N-formylmethionyl-leucyl-phenylalanine* (*fMLP) signalling in neutrophils, production of nitric oxide and reactive oxygen species in macrophages* and *vitamin D receptor activation.* In contrast, no key COPD pathways were identified that are associated with consistent actual upregulation and/or predicted activation of downstream inflammatory mediators.

Consistent with the canonical pathway functional enrichment analysis, in sputum the most common terms associated with the top 10 significant gene ontology (GO) biological processes and molecular mechanisms were immune response, programmed cell death and cytokine signalling for both doses (Table S2). Overall, 347 and 409 GO biological processes and 11 and 26 GO molecular functions were found to be significantly affected (pFDR< 0.05) by treatment with CHF6001 800 and 1600 μg BID, respectively.

### Inflammatory gene interaction network analysis

**Fig. 3:**
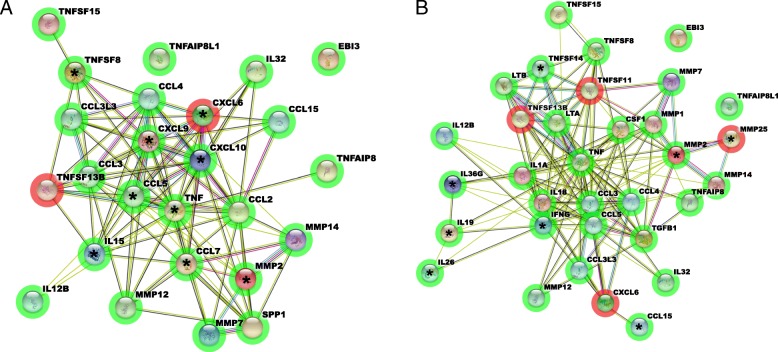
Network of pro-inflammatory cytokines and matrix metalloproteinases differentially expressed after treatment with CHF6001 A) 800 μg and B) 1600 μg BID relative to placebo. Each node represents all the proteins produced by a single, protein-coding gene locus, edges represent proteins that jointly contribute to a shared function, and the information inside the circle describes protein structure. Edges: a red line indicates the presence of fusion evidence; a green line, neighbourhood evidence; a blue line, co-occurrence evidence; a magenta line, experimental evidence; a yellow line, text mining evidence; a light blue line, database evidence; a black line, co-expression evidence; a purple line, protein homology evidence. A green halo around the nodes: downregulation, a red halo: upregulation. Unmarked nodes: pFDR< 0.05; * on the nodes: *p* < 0.05 and |FC| > 1.3

Gene networks comprising all pro-inflammatory cytokines and matrix-metalloproteinases differentially expressed after treatment with CHF6001 relative to placebo (pFDR< 0.05 or *p* < 0.05 and |FC| > 1.3) are shown in Fig. 3a and b. CHF6001 800 μg BID differentially expressed 25 pro-inflammatory cytokines and matrix metalloproteinases (16 with pFDR< 0.05 and nine with p < 0.05 and |FC| > 1.3), 23 of which were downregulated (Table S3). CHF6001 1600 μg BID differentially expressed 33 pro-inflammatory cytokines and matrix metalloproteinases (25 pFDR< 0.05 and eight with p < 0.05 and |FC| > 1.3), 29 of which were downregulated (Table S4). Most of the differentially expressed genes for CHF6001 800 μg BID were also differentially expressed for CHF6001 1600 μg BID (19 out of 25 genes, 76%), with all common genes differentially regulated in the same direction. The gene with the most network interactions for both CHF6001 doses was tumour necrosis factor (TNF; 20 interactions with 800 μg BID and 28 with 1600 μg BID); notably this was the only inflammatory mediator with direct interaction with the PDE4 genes (Fig. S4).

**Fig. 4:**
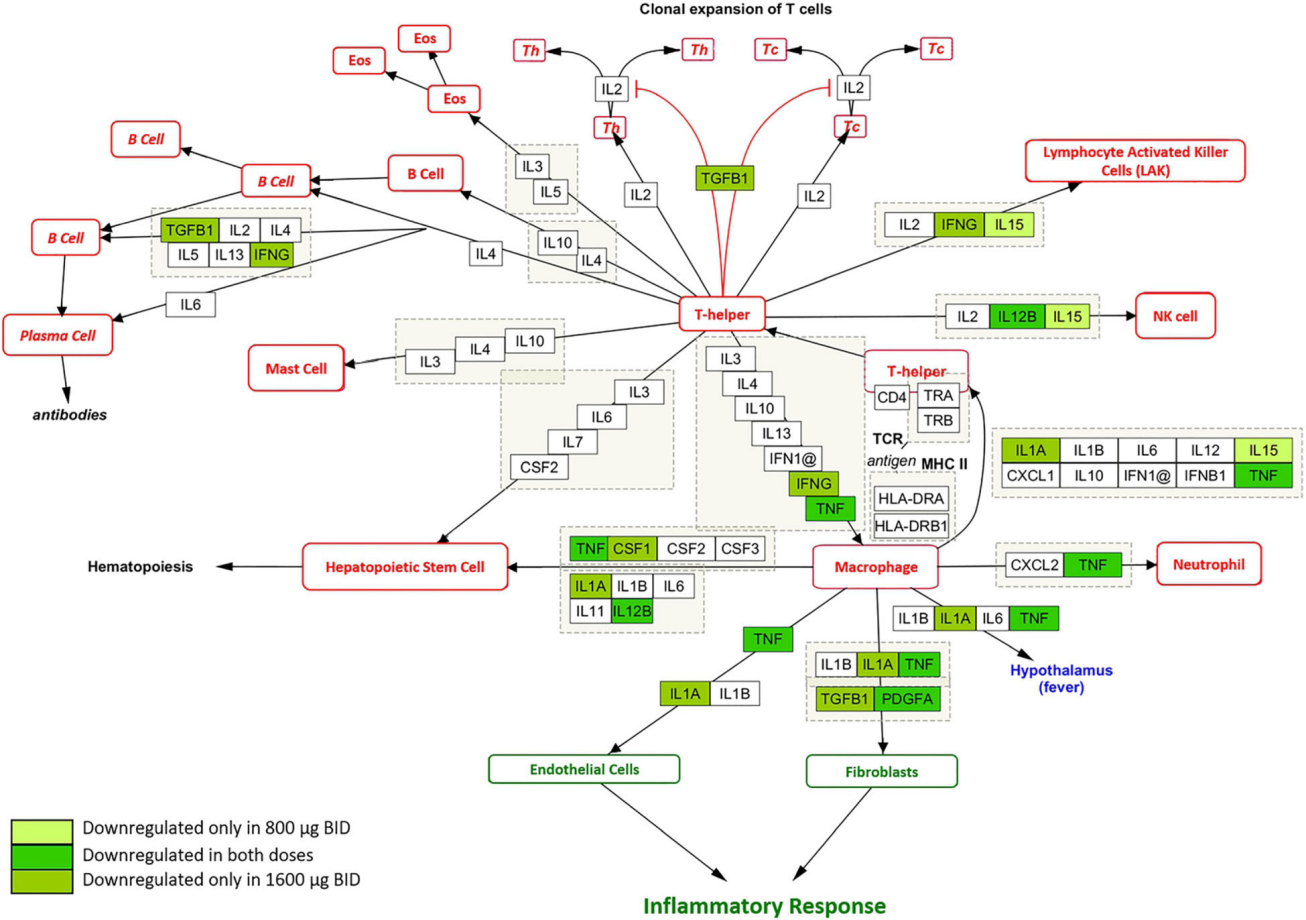
Cytokines and inflammation response from WikiPathways. Downregulated genes are coloured in different shades of green

In addition to the network and pathway analyses, WikiPathways [14] was used to visualise the biological pathways affected by differentially regulated genes. In particular, the pathway “Cytokines and Inflammatory Response (*Homo sapiens*)” was used to match the genes that were differentially expressed with both doses (i.e. pFDR< 0.05 or *p* < 0.05 and |FC| > 1.3). Only downregulated genes matched the pathway (Fig. 4): One was downregulated with CHF6001 800 μg BID (IL-15), four with CHF6001 1600 μg BID (transforming growth factor β1, [TGFB1], colony stimulating factor 1 [CSF1], interferon γ and IL-1A), and three with both CHF6001 doses (TNF, platelet-derived growth factor alpha polypeptide [PDGFA], and IL-12B).

Notably, CHF6001 significantly reduced the expression of many inflammatory genes known to be involved in the pathophysiology of COPD (Fig. S5, Table S5, Fig. S6 and Table S6). In particular, both doses led to a significant (pFDR< 0.05) downregulation of genes coding for pro-inflammatory TNF superfamily members, interferon gamma receptor (IFNGR2) [15], purinergic receptor (P2RX7) [16], endothelin 1 (EDN1) [17, 18], complement system (C3) [19, 20] and the profibrotics SERPINE1 [21] and platelet-derived growth factor alpha and beta (PDGFA, PDGFB) [20]. Furthermore, both doses also led to significant upregulation of the gene coding for the anti-inflammatory interleukin 10 receptor (IL-10RB) [15] and of the suppressor of cytokine signalling 3 (SOCS3) [22], and significantly modulated genes toward a positive regulation of the vitamin D pathway [23–27] (upregulation of VDR, RXR, DHCR7) and toward a negative regulation of oxidative stress [23, 28, 29] (upregulation of SESN2, HP, CYGB and downregulation of ATG7, CD1B, NCF1), Th2 cytokine production [23, 30] (upregulation of SCGB1A1 and TNFRSF21), B cells activation/proliferation [23, 31] (upregulation of SAMSN1, INPP5D) and significant downregulation of the eosinophil major basic protein (PRG2) [32]. With both doses, genes associated with eosinophils and basophils were significantly downregulated (CLC, OLIG1, OLIG2 and PRSS33) [33]. Treatment with the highest dose also significantly downregulated genes coding for the pro-inflammatory and mucus producer mitogen-activated protein kinase 13 (MAPK13) [23, 34], the cytotoxicity natural killer cell triggering receptor NCR3 [23], significantly upregulated the metalloproteinase inhibitor TIMP1 [23, 35] and the neutrophil elastase inhibitor SERPINB1 [36], and significantly modulated genes toward positive regulation of ciliary function (IFT88, DNAI2, DNAI1) [23, 37, 38].

## Discussion

**Fig. 5:**
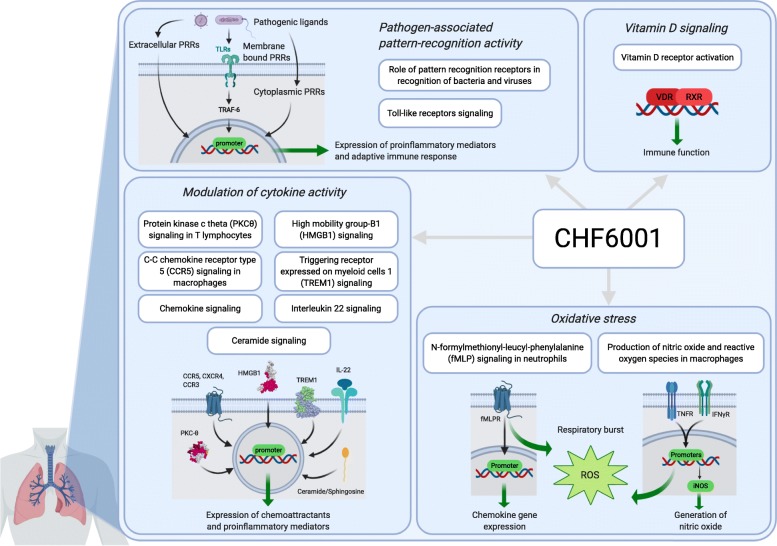
Key pathophysiological pathways significantly modulated by CHF6001 in sputum with associated inhibition of downstream inflammatory effectors. Created with Biorender.com

Here we show that CHF6001, on top of inhaled maintenance triple therapy, significantly modulated key pathophysiological inflammatory processes in sputum, specifically cytokine and matrix metalloprotease (MMP) activity, pathogen-associated pattern-recognition activity, oxidative stress and vitamin D (Fig. 5), demonstrating an add-on biological effect in the lung. In contrast, CHF6001 showed a non-significant pharmacodynamic effect in the blood. Mechanistically, these findings support the potential for CHF6001 to provide an additional beneficial effect in patients with chronic bronchitis who are still symptomatic despite regular use of ICS/LABA/LAMA, with a favourable systemic tolerability profile.

The functional enrichment analysis in sputum cells highlighted an effect of CHF6001 on growth, development, inflammation, immune response and cytokine signalling processes. The majority of significant pathways modulated by CHF6001 are associated with the pathophysiology of COPD including cytokines, cAMP, Th1, Th2, dendritic cells, natural killer cells, oxidative stress, and vitamin D pathways. Furthermore, cytokines and MMPs play a major role in the regulation of immune response and inflammatory conditions [15, 39]. An analysis of the differentially regulated genes in our database clearly showed that the number of downregulated inflammatory cytokines and MMPs outweighed the number of those upregulated (92 and 88% of the differentially regulated genes were downregulated with CHF6001 800 and 1600 μg BID, respectively). In addition, the canonical pathway analysis showed significant modulation associated with actual downregulation and/or predicted inhibition of downstream inflammatory mediators for key inflammatory pathways involved in cytokine activity (specifically CCR5 [40], chemokine, high mobility group-B1 [41], TREM1 [42], IL-22 [15], ceramide [43] and PKCθ signalling [44]), pathogen-associated pattern-recognition activity (pattern recognition receptors for the recognition of bacteria [45, 46] and viruses and toll-like receptors signalling [47]), oxidative stress (fMLP signalling in neutrophils [48], and nitric oxide and reactive oxygen species production in macrophages [29]), and vitamin D signalling [25–27].

Importantly, analysis of canonical pathways was carried out using a mixed model of actual and/or predicted regulation to simulate the downstream consequences of molecules mediated by the treatment. This simulation showed a straightforward concordance with the actual downregulation of pro-inflammatory cytokines and MMPs (Fig. 3) which comprises most of the downstream effectors of each identified pathway (Fig. S3).

The trend toward decreased inflammation was apparent in the WikiPathway analyses, in which eight differentially expressed genes in our database matched the pro-inflammatory mediators included in the pathways, all of which were downregulated by both CHF6001 doses.

Among the pro-inflammatory cytokine genes significantly differentially expressed, IL-32, IL-12B, EBI3 (IL-27B protein), TNFAIP8 (tumour necrosis factor alpha-induced-protein-8), TNFSF15 (tumour necrosis factor ligand-superfamily-member-15), CCL3 (MIP-α) and CCL4 (MIP-1β) were downregulated with both CHF6001 doses. Importantly, IL-12, IL-32 and IL-27 induce a variety of inflammatory signalling, and are linked to IFN-γ which is the predominant cytokine produced by Th1 and Tc1 cells and plays a role in inflammation in COPD [15, 49, 50]. Notably the IFN-γ gene was downregulated by the lowest dose (*p* < 0.05, |FC| > 1.3), as were CXCL10 (p < 0.05, |FC| > 1.3) and SPP1 (osteopontin protein), which enhance production of IFN-γ and IL-12 [51, 52]. Furthermore, expression of CCL3 and CCL4 are elevated in the lungs of individuals with COPD, both of which code for cytokines that activate CCR5 receptors and contribute to the recruitment of macrophage and T cells into the airways [15]. Interestingly, CCR5 (significantly downregulated with the lower dose) and its ligand CCL5 (RANTES proteins; significantly downregulated with the higher dose) are increased in airways and sputum of patients with COPD during exacerbations [15]. Other key cytokine genes significantly downregulated by CHF6001 were CCL15 for the lower dose, which is a potent inflammatory cell chemotactic agent, and, for the high dose, IL-1A, TNF, IL-18, CSF1, TGFB1, LTA (lymphotoxin-alpha or tumour necrosis factor-beta protein), and LTB (lymphotoxin-beta or tumour necrosis factor C protein). Proteins coding for these genes are involved in chemotaxis, positive regulation of cytokine activity, chronic inflammation and oxidative stress [15, 53, 54]. CHF6001 also significantly downregulated a wide range of metalloprotease genes (MMP7, MMP12, MMP14, and, with the higher dose, MMP1) which cause morphological changes in the lungs and contribute significantly to the COPD state [25–27].

Since the unbiased analysis of all the differentially expressed inflammatory cytokines and MMPs in our dataset was used as a tool to probe the overall level of inflammation after treatment in sputum, it is important to recognise that the effect was not consistent for all the mediators. Indeed, the gene coding for the chemokine (C-X-C motif) ligand 6 (CXCL6), which is a chemoattractant for neutrophilic granulocytes, and the tumor necrosis factor ligand superfamily member 13B (TNFSF13B), which acts as a potent B cell activator, did not follow the same direction as the other pro-inflammatory mediators, being consistently upregulated with both doses of CHF6001.

Importantly, in this study we show a remarkable consistency between doses. Most of the probe sets significantly differentially expressed with the low dose were also differentially expressed with the high dose in the same direction, i.e. either both up-regulated or both down-regulated. In addition, we demonstrated a clear dose-response relationship both for the number of probe sets significantly modulated by CHF6001 and the differentially expressed fold change effect size.

Overall, the current results are in agreement with the analysis of biomarker data from these patients, showing that CHF6001 significantly decreased the levels of various target soluble inflammatory mediators in the sputum [10], namely leukotriene B4, C-X-C motif chemokine ligand 8, macrophage inflammatory protein 1β, matrix metalloproteinase 9, and tumour necrosis factor α (TNFα). The current analyses build on these findings, by providing a complete pharmacodynamic characterisation of the biological effect of CHF6001 in sputum cells and whole blood, covering nearly the whole genome coding for proteins. This approach provides an holistic picture of complex anti-inflammatory effects, which would be otherwise missed when measuring target biomarkers of inflammation at protein level.

To our knowledge this is the first ‘omics’ high-throughput placebo controlled biologic characterisation of an anti-inflammatory agent in COPD in the airways and systemic compartment. In addition, differentially regulated genes, pathways, biological processes and molecular functions were considered significant only upon correction for multiplicity (pFDR< 0.05). Few previous studies have investigated gene expression by microarray in blood after treatment of COPD patients with anti-inflammatory agents [33, 55, 56] and most of them did not correct for multiplicity [55, 56]. One study evaluated the effect of azithromycin by microarray both in blood and sputum of COPD patients, although the analyses were not corrected for placebo [57]. Taken together, these findings highlight the difficulties in generating significant results in complex transcriptomics studies, in which correction for multiplicity for thousands of concomitant assessments and the high variability makes obtaining statistically significant results prohibitive. To decrease variability, our trial was conducted in highly standardised conditions in a crossover design with an adequate washout to ensure there was no carry-over effect between the different periods. All clinical sites were highly skilled with blood and sputum collection and processing, leading to an average viability of sputum cells of 92.5%. In addition, the quality control analysis highlighted the high quality of RNA, with 100% of the blood samples and 99.3% of the sputum samples being acceptable. We acknowledge that the analyses have some limitations. A small number of matching pre-dose samples were replaced with the pre-dose samples from the closest available period, assuming there was no period effect on pre-dose expression. We recognise that this might increase uncertainty on the robustness of our results, but the validity of this assumption was supported by i) the lack of significant associations in the principal component analysis between pre-dose gene expression data and period/treatment; ii) the lack of significant differences between treatments in pre-dose expression for any of the analysed probe sets (see ANOVA analysis in supplement, results section); and iii) the high concordance of study results between different doses of CHF6001.

## Conclusions

The current analyses demonstrate that in patients with chronic bronchitis receiving maintenance inhaled triple therapy, CHF6001 inhaled BID significantly modulates immune system processes in sputum but not in blood. Canonical pathway and network analyses highlight downregulation of key pro-inflammatory pathways and mediators involved in the pathophysiology of COPD in sputum. This is the profile that CHF6001 was designed to demonstrate: extrafine inhaled administration with high systemic clearance and high protein binding to minimise the pharmacodynamic effect in blood (and thus optimise systemic safety) and maximise the biological effect in the lung.

## Supplementary information

**Additional file 1.** Supplementary methods and results. Methods and results supporting main body of the manuscript

## Data Availability

Chiesi commits to sharing with qualified scientific and medical Researchers, conducting legitimate research, patient-level data**,** study-level data**,** the clinical protocol and the full clinical study report of Chiesi Farmaceutici S.p.A.-sponsored interventional clinical trials in patients for medicines and indications approved by the European Medicines Agency and/or the US Food and Drug Administration after *1st January 2015*, following the approval of any received research proposal and the signature of a Data Sharing Agreement. Chiesi provides access to clinical trial information consistently with the principle of safeguarding commercially confidential information and patient privacy. To date, the current study is out of scope of the Chiesi policy on Clinical Data Sharing. Other information on Chiesi’s data sharing commitment, access and research request’s approval process are available in the Clinical Trial Transparency section of http://www.chiesi.com/en/research-and-development/.
