# Supplementary material for: Sputum and blood transcriptomics characterisation of the inhaled PDE4 inhibitor CHF6001 on top of triple therapy in patients with chronic bronchitis
Source: Respir Res. 2020 Mar 20;21:72. doi: 10.1186/s12931-020-1329-y (PMC7085203; doi:10.1186/s12931-020-1329-y)
Supplement: Supplementary file 1 — Additional file 1. Supplementary methods and results. Methods and results supporting main body of the manuscript [file 12931_2020_1329_MOESM1_ESM.pdf]

Sputum and blood transcriptomics characterisation of the inhaled PDE4 inhibitor CHF6001 in COPD

Mirco Govoni, Michele Bassi, Stefano Vezzoli, Germano Lucci, Aida Emirova, Marie Anna Nandeuil, Stefano Petruzzelli, Gera Jellema, Ebenezer Alfolabi, Brendan Colgan, Brian Leaker, Oliver Kornmann, Kai Michael Beeh, Henrik Watz, Dave Singh

Supplement

Supplementary methods

Ethics committees

The study was approved by two centralised ethics committees on behalf of the sites:

- UK: Health Research Authority, North West, Greater Manchester South Research Ethics Committee, Manchester (approval reference 16/NW/0553).
- Germany: Ethics Committee of Hesse Medical Association, Frankfurt am Main (approval reference FF 98/2016).

Inclusion Criteria

Patients had to meet all the following inclusion criteria to be eligible for enrolment into the study:

1. Written informed consent obtained prior to any study-related procedures;
2. Male or female aged ≥40 years;
3. A female was eligible to enter the study if she was of non-childbearing potential, i.e. physiologically incapable of becoming pregnant (e.g. postmenopausal women defined as being amenorrhoeic for ≥12 consecutive months without an alternative medical cause. If indicated, as per Investigator's request, post-menopausal status was confirmed by analysis of follicle-stimulating hormone levels, according to local laboratory ranges) or women permanently sterilised (e.g. bilateral oophorectomy, hysterectomy or bilateral salpingectomy). Women physiologically capable of becoming pregnant (i.e. women of childbearing potential) were eligible to enter the study if they had a negative pregnancy test at screening and agreed to use one or more of the following highly effective contraceptive measures:
    a. Placement of an intrauterine device or intrauterine hormone-releasing system;
    b. Combined (containing both oestrogen and progestogen) hormonal contraception associated with inhibition of ovulation (oral, intravaginal, transdermal);

   c. Progesterone-only hormonal contraception associated with inhibition of ovulation (oral, injectable, implantable);
   d. Bilateral tubal occlusion;
   e. Vasectomised partner.

   Reliable contraception had to be maintained throughout the study.

   Abstinence was acceptable when in line with the patient’s preferred and usual lifestyle. A pregnancy serum test was performed in all women of childbearing potential at screening and at Day 32 of Period 3. A pregnancy urine test was performed in all women of childbearing potential at Day 1 and Day 32 of each treatment period and at the follow-up visit;

4. Patients with an established diagnosis of COPD (according to Global Initiative for Chronic Obstructive Lung Disease, 2015) at least 12 months prior to the screening visit;
5. A smoking history of at least 10 pack-years [pack-years = number of cigarettes per day x number of years/20]. Current and ex-smokers were eligible (smoking cessation was at least three months prior to the screening. If the patients underwent smoking cessation therapy, it must have been completed three months prior to the screening visit);
6. A body-mass index in the range of 18–35 kg/m^2;
7. A post-bronchodilator forced expiratory volume in 1 second (FEV_1) ≥30% and ≤70% of the patient normal predicted value and a post-bronchodilator FEV_1/forced vital capacity (FVC) ratio <0.70 measured 10–15 minutes after 400 µg (4 puffs x 100 µg) of salbutamol via pressurised metered-dose inhaler pMDI. If this criterion was not met at screening, the test was repeated once before the randomisation visit;
8. Patients must have been receiving daily maintenance with triple therapy (inhaled corticosteroid [ICS] plus long-acting muscarinic antagonist [LAMA] plus long-acting β_2-agonist [LABA]) at a stable dose and dosing regimen for at least two months prior to screening;

9. A history of chronic bronchitis defined as chronic cough and sputum production for more than three months per year for two or more years and known as a ‘spontaneous sputum producer’;
10. At screening, patients must have been able to produce an adequate induced sputum sample defined as a load of at least 300 mg with a viability factor of not less than 70% (with less than 30% epithelial cells) and a neutrophil % differential count of at least 60%. The patients may have been re-challenged once, if the first sputum sample did not meet these criteria;
11. Patients must have been symptomatic at screening, defined as having a COPD Assessment Test score ≥10;
12. Patients had to be able to be trained to use the DPI inhalers (NEXThaler®) correctly and to generate sufficient peak inspiratory flow (PIF) (at least 40 L/minute) using the In-Check Dial® device;
13. A cooperative attitude and ability to perform the required outcome measurements (e.g. spirometry testing, induced sputum, and other analyses).

Exclusion Criteria

The presence of any of the following excluded a patient from study enrolment:

1. Pregnant or lactating women;
2. Patients with a current diagnosis of asthma;
3. Patients with a moderate or severe COPD exacerbation (i.e. resulting in the use of systemic [oral/intravenous [IV]/intramuscular [IM] corticosteroids] and/or antibiotics or in hospitalisation) or a lower respiratory tract infection within 6 weeks prior to study entry or during the screening period;
4. Patients on maintenance bronchodilators therapy only (LABA alone, LAMA alone, dual LABA/LAMA alone) or maintenance dual therapy only (ICS/LABA or ICS plus LAMA) within two months prior to study entry;
5. Patients on phosphodiesterase (PDE) 4 inhibitors (e.g. roflumilast) within two months prior to study entry;

6. Patients requiring long-term (at least 12 hours daily) oxygen therapy for chronic hypoxemia;
7. Patients participating in a pulmonary rehabilitation program or completing such a program within the last six weeks prior to study entry;
8. Patients with known respiratory disorders other than COPD that in the Investigator’s opinion would affect efficacy and safety evaluation or place the patient at risk. This included, but was not limited to, known α-1 antitrypsin deficiency, active tuberculosis, bronchiectasis, sarcoidosis, lung fibrosis, pulmonary hypertension and interstitial lung disease;
9. Patients with lung cancer or a history of lung cancer;
10. Patients with active cancer or a history of cancer (other than lung) with less than five years disease-free survival time (whether there was evidence of local recurrence or metastases or not). Localised carcinoma (e.g. basal cell carcinoma [without metastases], in situ carcinoma of the cervix adequately treated) was acceptable;
11. Patients with a known history of hypersensitivity to β_2-agonist, PDE4 inhibitors or any of the excipients contained in any of the formulations used in the study;
12. Patients with a diagnosis of depression associated with suicidal ideation or behaviour or with a diagnosis of generalised anxiety disorder that in the Investigator’s opinion would place the patient at risk;
13. Patients who had known history of clinically significant (CS) cardiovascular conditions such as, but not limited to, unstable or acute ischemic heart disease within one year prior to study entry, New York Heart Association Class III/IV heart failure, known history of sustained and non-sustained cardiac arrhythmias or history of atrial fibrillation diagnosed in the last 6 months prior to study entry and not controlled with therapy rate control strategy;
14. Patients who had CS abnormal 12-lead ECG that, in the Investigator’s opinion, would affect efficacy or safety evaluation or place the patient at risk;

15. Male patients with a Fridericia-corrected time interval between the Q and T waves (QT) (QTcF) > 450 ms and female patients with a QTcF > 470 ms at screening and/or at randomisation visits;
16. Patients with a history or symptoms of significant neurological disease including transient ischemic attack, stroke, seizure disorder or behavioural disturbances;
17. Patients with unstable concurrent disease: e.g. uncontrolled hyperthyroidism; uncontrolled diabetes mellitus or other endocrine disease; significant hepatic impairment; significant renal impairment; history of cerebrovascular disease; uncontrolled gastrointestinal disease (e.g. active peptic ulcer, Crohn’s disease, ulcerative colitis, enteritis, unexplained diarrhoea, bloody or loose stools); uncontrolled haematological disease; uncontrolled autoimmune disorders (e.g. rheumatoid arthritis, inflammatory bowel disease) or other disease or condition that might, in the judgement of the Investigator, place the patient at undue risk or potentially compromise the results or interpretation of the study;
18. Patients with CS laboratory abnormalities indicating a significant or unstable concomitant disease that might, in the judgement of the Investigator, place the patient at undue risk or potentially compromise the results or interpretation of the study;
19. Patients with abnormal alanine aminotransferase (ALT) ≥2x upper limit of normal (ULN) and/or aspartate aminotransferase (AST) ≥2x ULN and/or bilirubin ≥1.5x ULN. Isolated bilirubin ≥1.5x ULN was acceptable if fractionated and direct bilirubin was <35%;
20. Current or chronic history of liver disease, or known hepatic or biliary abnormalities (except for Gilbert’s syndrome or asymptomatic gallstones);
21. Patients receiving treatment with any drug known to have a well-defined potential for hepatotoxicity (e.g. isoniazid, nimesulide, ketoconazole) within the previous three months prior to study entry and during the screening period;
22. Patients who experienced excessive weight loss recently (which could not be explained by the natural course of COPD or known background conditions);

23. Patients with a history of alcohol abuse and/or substance/drug abuse within 12 months prior to the screening visit;
24. Patients that received any other investigational drug within the preceding 30 days (60 days for biologics), or a longer and more appropriate time as determined by the Investigator (e.g. approximately five half-lives of the previous investigational drug).

Sputum collection and processing for RNA assessments

Sputum samples were collected by sputum induction, with patients breathing in nebulised hypertonic saline at increasing concentrations (3%, then 4% and finally 5%) while wearing a nose clip.

All solid or dense-looking material was collected from the clear salivary fluid into a stainless steel Petri dish lid. Sputum plugs were then gathered and condensed into one mass. After cleaning the Petri dish, cold Dulbecco's Phosphate-Buffered Saline equal to eight times the weight of the sputum plug was added, then dispersed, mixed and vortexed for 15 sec, before gently agitating on a rolling mixer or rocker for 15 min at room temperature. The resulting mixture was centrifuged at 790 g for 10 min at 4C. A volume of the supernatant equal to four times the weight of the sputum plug was replaced with an equivalent volume of Sputolysin 0.2% and gently agitated on a rolling mixer or rocker for 15 min at room temperature. The resulting cell suspension was filtered, with the filtrate centrifuged at 790 g for 10 min at 4C, and the supernatant was removed. The pellet was re-suspended in cold Dulbecco's Phosphate-Buffered Saline for total and differential cell count, centrifuged at 400g for 10 minutes at 4C and re-suspend again in TRIzol to give approximately 1x106 cells for 1mL of TRIzol for RNA assessment. The resulting suspension were stored immediately in an upright position at -80°C until shipment to the laboratory on dry ice for RNA extraction and microarray analysis.

Blood collection and processing for RNA assessment

Blood was collected in BD Vacutainer™ PAXgene™ Blood RNA tubes suitable for storage and transportation of whole blood that requires intracellular RNA stabilisation. Empty PAXgene tubes were stored at nominally +4C and kept upright at room temperature (+18C to +25C) for a minimum of 2 hours and a maximum of 72 hours prior to use. A few mL of blood were discarded from the catheter prior to drawing blood into the PAXgene Blood RNA. After collection tubes were inverted 8-10 times and stored into a –20C freezer for at least 24 hours and then transferred into a –80C freezer until shipment to be bioanalytical lab in dry ice where they were stored at –80C until RNA extraction and analysis.

RNA extraction and amplification

RNA was extracted from the Sputum TRIzol/lysate using a standard phenol/chloroform extraction method. This method combines a robust lysis/denaturant extraction with ethanol precipitation for recovery of high-quality total RNA. The crude RNA was further purified by incubation with DNaseI (Qiagen, Hilden, Germany) followed by filter purification using the QIAgen RNeasy MinElute spin column (Qiagen, Hilden, Germany) to yield pure RNA.

RNA was extracted from the blood samples using the PAXgene™ Blood RNA Kit on the automated QIAsymphony SP module (Qiagen, Hilden, Germany). Blood samples were left to thaw at room temperature for at least 2 h before commencing the RNA extraction protocol. Following equilibration at room temperature, samples were centrifuged to pellet the nucleic acids. The pellets were re-suspended and incubated with Proteinase K in a buffer containing guanidine-isothiocyanate. The samples were inserted in the QIAsymphony SP module (Qiagen, Hilden, Germany) where the RNA

was purified using magnetic particle processing. Following elution the RNA was heat denatured at 65C.

The extracted RNA samples were then assessed for concentration and purity using the Nanodrop 1000 spectrophotometer (Thermo Fisher Scientific, Massachusetts, USA). Quality was assessed using the Agilent Bioanalyser 2100 system (Agilent, Santa Clara, USA). RNA samples were reverse transcribed and amplified using the NuGEN™ Ovation™ RNA Amplification System V2 (NuGen Technologies, Redwood City, California) in combination with the Ovation™ Whole Blood Solution, and quality assessed again. The amplified ssDNA was then fragmented and labelled using the NuGEN Encore Biotin Module (NuGen Technologies, Redwood City, California), before being hybridised onto Affymetrix GeneChip® (Human Plus 2.0) arrays (Thermo Fisher Scientific, Santa Clara, California).

Sample profiling

All samples with sufficient quality (two identifiable peaks at 18S and 28S) and quantity (sputum 20 ng input and blood 50 ng input) of RNA were profiled on Affymetrix U133 Plus 2.0 microarrays. Prior to profiling, samples were randomised into batches for microarray analysis (amplification and array lot) according to available clinical and technical factors (centre, data of test, stage, treatment period and sex)

Microarray data quality control and pre-processing

Probe-level intensity measurements (CEL files) were background corrected, normalised and summarised as expression measurements using Robust Multichip Algorithm (RMA) [1] within each dataset (blood, sputum) separately. Data quality control was performed using Affymetrix GeneChip QC parameters, array image quality analysis and distribution analyses, according to Almac Diagnostic Services

QC pipeline. Surface artefacts were identified by modelling the expected variation in signal intensity over the array in order to find areas of unexpectedly high deviation; outlying Gene Chip QC parameters were identified using absolute deviation from the measure of central tendency; samples with outlying intensity profiles were identified using PCA (Hoteling T2 and Residual Q). Before further analysis, samples that passed quality assessment were pre-processed again using RMA. The data were subsequently filtered to remove any uninformative transcripts (lowly expressed, invariant probe sets). Probe sets were removed using a background variance measure defined from the data.

In advance of identification of differentially expressed genes, as part of the data quality assessment, the impact of RNA-integrity number (RIN) on resultant data was observed. Although sample selection for profiling included selection of samples with RINs within the tolerance of the amplification system, it was considered pertinent to confirm that the resulting data quality was not influenced by variability in sample degradation within the cohort. The profiling chemistry and array platform are designed to maximise amplification & measurement of transcripts present within the sample, and the aim of the data normalisation is to minimise variation due to differences in input quantity. However, despite best attempts to generate comparable data, stark differences in input sample quality will cause differences in resulting data quality. Percentage present call is a measure of detection rate of transcripts targeted by the probe sets on the microarray; it is the primary measure of data quality and is reflective of sample quality. In this cohort, no relationship between RIN and percentage present call was found (figure provided if required, correlation may be sufficient).

Exclusion of probe sets with statistically significantly different pre-dose expression between treatment periods

An ANOVA model was fitted to identify probe sets with statistically significantly different pre-dose expression between treatment periods, with pre-dose expression as dependent variable, and subject, period and treatment as independent variables. In case of significant differences (pFDR <0.05 for the comparisons between CHF6001 doses and placebo), the probe set was excluded from further analysis.

Results

Total and differential cell count in sputum

Figure S1. Change from baseline (average of Day 20, 26 and 32) geometric means ratio of CHF6001 relative to placebo for total cell count, and absolute and relative differential cell counts in sputum (analysed population).

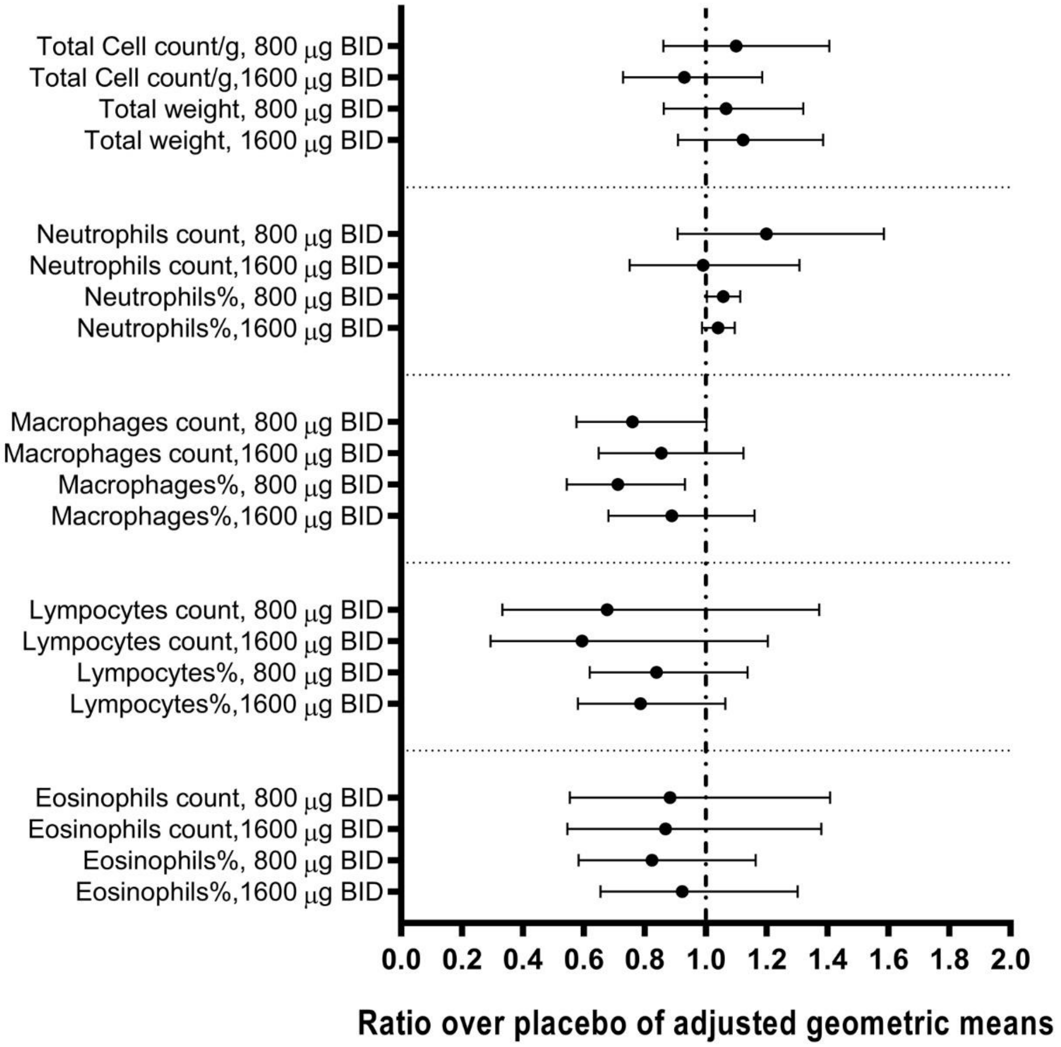

Abbreviations: BID, twice daily. Data are the ratios of geometric means and 95% CI.

Samples

Following quality and RNA quantity assessment in blood, 51 post-dose samples for placebo, 54 for CHF6001 800 µg BID, and 55 for CHF6001 1600 µg BID were available for analysis. Among the pre-dose samples considered for analysis, six for placebo, six for CHF6001 800 µg BID and 10 for CHF6001 1600 µg BID did not

match the corresponding post-dose of the same periods. Overall, pre- to post-dose samples for active and placebo were available from 50 patients for both CHF6001 treatments.

Following quality and RNA quantity assessment in sputum, 43 post-dose samples (33 on Day 32 and 10 on Day 26) were available for placebo, 41 samples (30 on Day 32, seven on Day 26, and four on Day 21) were available for CHF6001 800 µg BID and 41 samples (35 on Day 32, five on Day 26, and one on Day 21) were available for CHF6001 1600 µg BID. Among the pre-dose samples, 13 for placebo, six for CHF6001 800 µg BID and three for 1600 µg BID did not match the corresponding post-dose of the same periods. Overall, pre- to post-dose samples for active and placebo were available from 37 patients for CHF6001 800 µg BID and 41 patients for CHF6001 1600 µg BID.

Principal component analysis on pre-dose samples

Analysis of principal components on pre-dose samples both in blood and sputum (Figure S2).

Figure S2. PCA image of all sputum (A, B) and blood (C, D) pre-dose samples, coloured by period (A, C) or by treatment (B, D)

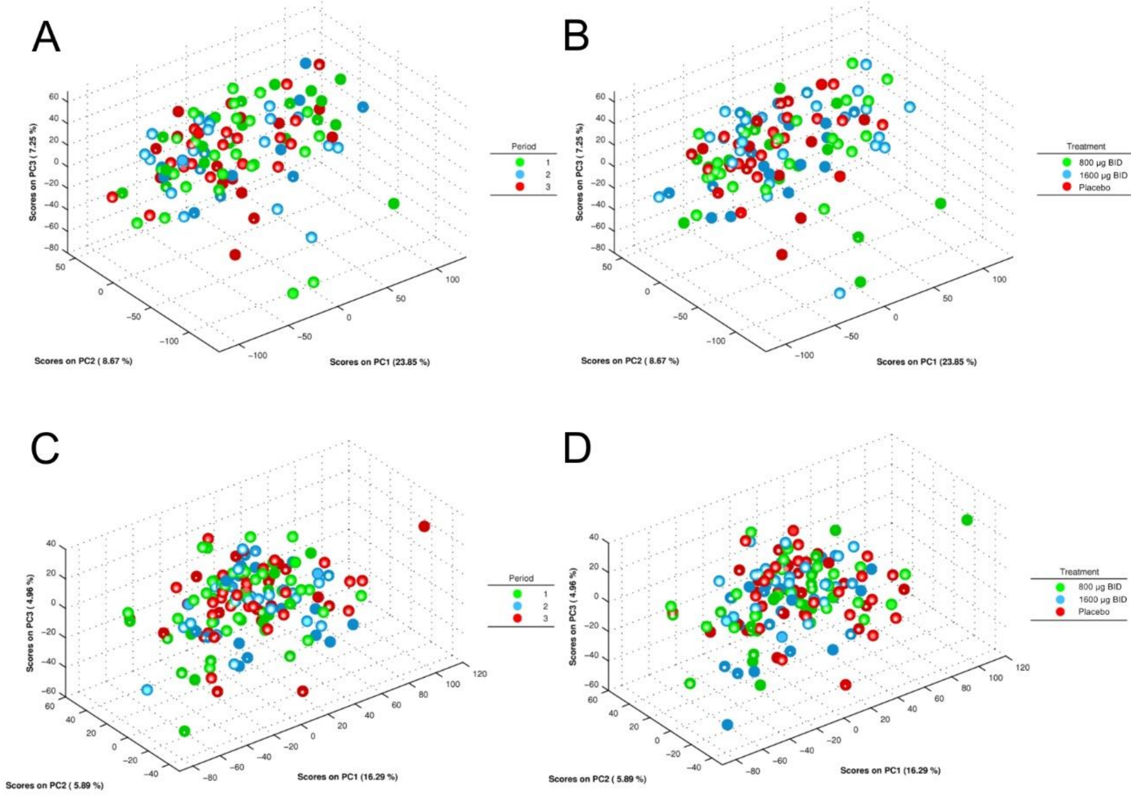

Microarray data quality control and pre-processing

More than 54,000 probe sets were considered, corresponding to approximately 21,000 genes coding for proteins. Filtering to remove any uninformative transcripts (low expression, invariant probe sets) using a background variance of 3.49 for blood and 3.60 for sputum resulted in 45,163 and 44,355 reliably detected probe sets in blood and sputum, respectively, available for analysis.

Functional enrichment analysis

Table S1: Canonical pathways significantly modulated by CHF6001. *******Associated with the pathophysiology of COPD; †Probability adjusted for multiple test correction of a random gene set this size to co-concur on this group; ‡Molecules from the Gene list that are annotated to the functional group*

**Ingenuity canonical pathways**	**Association with COPD***	**CHF6001 800 µg BID**		**CHF6001 1600 µg BID**	
		**p-value (FDR)†**	**Genes‡**	**p-value (FDR)***	**Genes‡**
Macropinocytosis signalling	[2]	0.0324	PDGFB, PDGFA, PIK3CG, ITGB7, SRC, KL, PRKCZ, ANKFY1, HGF	0.0417	PDGFB, PLCG2, PDGFA, IRS2, CSF1, PIK3CG, KL, PRKCZ, PAK1
Platelet-derived growth factor (PGDF) signalling	[3]	0.0324	PDGFB, SPHK1, PDGFA, INPP5D, PIK3CG, MAP2K4, SRC, KL, SYNJ2	0.0038	PDGFB, PLCG2, PDGFA, IRS2, SYNJ1, JUN, INPP5D, PIK3CG, SOS2, MAP2K4, RASA1, KL, STAT3
Relaxin Signalling	[4]	0.0324	GNG2, PDE4D, PDE4B, PIK3CG, PRKAR1A, GNAT2, KL, PRKCZ, PDE7A, PDE2A, BRAF, GNB5	0.0117	PDE4D, JUN, PDE4B, PIK3CG, PRKAR1A, KL, GNG4, BRAF, GNB5, GNG2, IRS2, GNAI3, GNAT2, PRKCZ, PDE7A, PDE2A
Hepatic fibrosis / hepatic stellate cell activation		0.0324	PDGFB, IL-1RL2, PDGFA, BCL2, IGF1R, IL-18RAP, EDN1, MYH10, SERPINE1, IL-1RL1, COL1A1, IL-1R1, HGF	0.0038	IL-1RL2, IL-18RAP, MYH10, TLR4, IL-4R, COL1A1, CCL5, COL15A1, MMP1, PDGFB, PDGFA, CSF1, IGF1R, EDN1, IL-1A, FAS, SERPINE1, IL-1RL1, IL-1R1, TNF
Vitamin D receptor (VDR)/RXR activation	[5–7]	0.0324	VDR, RXRA, PDGFA, CST6, CDKN1B, CYP27B1, PRKCZ, IL-1RL1	0.0339	SERPINB1, PDGFA, CST6, CDKN1B, CYP27B1, TNFSF11, PRKCZ, IL-1RL1, CCL5

Ingenuity canonical pathways	Association with COPD*	CHF6001 800 µg BID		CHF6001 1600 µg BID	
		p-value (FDR)†	Genes‡	p-value (FDR)*	Genes‡
Signal transducer and activator of transcription 3 (STAT3) pathway	[8]	0.0324	PDGFB, BCL2, IL-27RA, IGF1R, MAP2K4, SOCS3, SRC, HGF, BMP6	NS	
Phosphatidylinositol 3,4,5-trisphosphate 3-phosphatase and dual-specificity protein phosphatase (PTEN) signalling	[9]	0.0324	CNKSR3, BCL2, IGF1R, INPP5D, PIK3CG, BCL2L11, CASP9, CDKN1B, PRKCZ, SYNJ2	NS	
G-protein coupled receptor signalling	[10]	0.0331	PDE4D, DUSP4, PDE4B, PIK3CG, PRKAR1A, MAP3K8, DUSP6, PTGIR, KL, BRAF, CXCR2, HTR2B, SRC, PDE7A, PDE2A, PTGER2	0.0162	PDE4D, DUSP4, PDE4B, PIK3CG, PRKAR1A, MAP3K8, SOS2, FPR2, DUSP6, KL, BRAF, RGS18, ADORA2A, IRS2, FPR1, GNAI3, CXCR2, RASA1, PDE7A, PDE2A, STAT3, PLCB1, PTGER2
1D-myo-inositol Hexakisphosphate Biosynthesis II (mammalian)	[3]	0.0331	INPP5A, INPP5D, IPMK, SYNJ2	NS	

Ingenuity canonical pathways	Association with COPD*	CHF6001 800 µg BID		CHF6001 1600 µg BID	
		**p-value (FDR)†**	**Genes‡**	**p-value (FDR)***	**Genes‡**
D-myo-inositol (1,3,4)-trisphosphate biosynthesis	[3]	0.0331	INPP5A, INPP5D, IPMK, SYNJ2	NS	
Cyclic adenosine monophosphate (cAMP)-mediated signalling	[11,12]	0.0331	PDE4D, DUSP4, PDE4B, PRKAR1A, CXCR2, DUSP6, PTGIR, SRC, PDE7A, AKAP12, PDE2A, PTGER2, CREM, BRAF	0.0145	PDE4D, DUSP4, PDE4B, PRKAR1A, FPR2, DUSP6, AKAP12, AKAP13, CREM, BRAF, RGS18, ADORA2A, FPR1, GNAI3, CXCR2, PDE7A, PDE2A, STAT3, PTGER2, CAMK1
Small cell lung cancer signalling		0.0331	KL, TFDP1, RXRA, BCL2, PIK3CG, CCNE2, CASP9, CDKN1B	NS	
Role of osteoblasts, osteoclasts and chondrocytes in rheumatoid arthritis		0.0355	IL-1RL2, MMP14, BCL2, PIK3CG, IL-18RAP, IL-18R1, MAP2K4, CASP9, KL, SRC, IL-1RL1, COL1A1, IL-1R1, BMP6	0.0038	IL-1RL2, JUN, IL-18, PIK3CG, IL-18RAP, KL, ALPL, TNFSF11, NFATC2, COL1A1, SMAD9, MMP1, BMP6, IRS2, CSF1, MAP2K4, IL-18R1, IL-1A, DKK2, SFRP1, IL-1RL1, IL-1R1, TNF
Human epidermal growth factor receptor 2 (HER-2) signalling in breast cancer		0.0355	KL, PRKCZ, AREG, PIK3CG, CCNE2, ITGB7, CASP9, CDKN1B	NS	

Ingenuity canonical pathways	Association with COPD*	CHF6001 800 µg BID		CHF6001 1600 µg BID	
		p-value (FDR)†	Genes‡	p-value (FDR)*	Genes‡
IL-10 signalling	[13]	0.0355	SOCS3, IL-1RL2, IL-1RL1, IL-18RAP, MAP2K4, IL-1R1, FCGR2A	<0.0001	IL-1RL2, JUN, IL-18, IL-18RAP, MAP2K4, FCGR2A, IL-1A, CCR1, SOCS3, IL-4R, IL-1RL1, MAP4K4, STAT3, IL-1R1, TNF
Myc mediated apoptosis signalling		0.0363	KL, PRKCZ, BCL2, IGF1R, PIK3CG, MAP2K4, CASP9	0.0490	IRS2, IGF1R, PIK3CG, SOS2, MAP2K4, KL, PRKCZ, FAS
Hepatic cholestasis		0.0363	IL-1RL2, RXRA, IL-12B, ABCB4, IL-18RAP, PRKAR1A, MAP2K4, PRKCZ, IL-1RL1, OSM, IL-1R1	0.0240	IL-1RL2, IL-12B, ABCB4, JUN, IL-18, IL-18RAP, PRKAR1A, MAP2K4, IL-1A, RARA, TLR4, PRKCZ, IL-1RL1, IL-1R1, TNF
PPAR signalling	[14]	0.0363	PDGFB, STAT5B, IL-1RL2, RXRA, PDGFA, IL-1RL1, IL-18RAP, IL-1R1	0.0038	STAT5B, PDGFB, IL-1RL2, PDGFA, JUN, IL-18, IL-18RAP, SOS2, IL-1A, IL-1RL1, MAP4K4, IL-1R1, TNF
Cysteine biosynthesis III (mammalian)	[15]	0.0363	CTH, MAT2A, EEF1AKMT3, NSUN4	NS	
Antiproliferative role of somatostatin receptor 2		0.0372	GNG2, KL, SRC, PIK3CG, BRAF, CDKN1B, GNB5	NS	

Ingenuity canonical pathways	Association with COPD*	CHF6001 800 µg BID		CHF6001 1600 µg BID	
		p-value (FDR)†	Genes‡	p-value (FDR)*	Genes‡
Tec kinase signalling	[16]	0.0398	STAT5B, GNG2, TNFRSF10B, PIK3CG, MAP2K4, GNAT2, SRC, KL, PRKCZ, BMX, GNB5	<0.0001	LCK, STAT4, PIK3CG, FNBP1, TLR4, KL, RHOF, PAK1, BMX, GNG4, GNB5, STAT5B, TNFRSF21, GNG2, PLCG2, IRS2, MAP2K4, GNAI3, GNAT2, RHOU, FAS, PRKCZ, STAT3, TNF
Superpathway of D-myo-inositol (1,4,5)-trisphosphate metabolism	[3]	0.0437	INPP5A, INPP5D, IPMK, SYNJ2	NS	
Role of macrophages, fibroblasts and endothelial cells in rheumatoid arthritis		0.0457	IL-1RL2, PIK3CG, IL-18RAP, IL-32, KL, SOCS3, PDGFB, PDGFA, MAP2K4, IL-18R1, TNFSF13B, SRC, PRKCZ, IL-1RL1, OSM, IL-1R1	0.0003	IL-1RL2, IL-18, JUN, IL-18RAP, PIK3CG, IL-32, TLR4, KL, SOCS3, TNFSF11, NFATC2, CCL5, MMP1, PLCG2, PDGFB, PDGFA, CSF1, IRS2, CEBPD, TNFSF13B, MAP2K4, LTB, IL-18R1, IL-1A, PRKCZ, DKK2, SFRP1, IL-1RL1, STAT3, PLCB1, IL-1R1, TNF
Role of JAK family kinases in IL-6-type cytokine signalling	[17]	0.0468	STAT5B, SOCS3, OSM, MAP2K4	NS	

Ingenuity canonical pathways	Association with COPD*	CHF6001 800 µg BID		CHF6001 1600 µg BID	
		**p-value (FDR)†**	**Genes‡**	**p-value (FDR)***	**Genes‡**
Th1 and Th2 activation pathway	[13,18]	NS		0.0004	JUN, IL-18, STAT4, PIK3CG, CRLF2, KL, SOCS3, IL-4R, TNFSF11, NFATC2, STAT5B, CD3E, IL-12B, IL-27RA, IRS2, IL-18R1, CCR1, CD3G, NFIL-3, KLRD1, IL-1RL1, STAT3
IL-6 signalling	[13]	NS		0.0008	IL-1RL2, MCL1, JUN, IL-18, PIK3CG, IL-18RAP, SOS2, KL, SOCS3, COL1A1, IRS2, MAP2K4, IL-1A, IL-1RL1, STAT3, MAP4K4, IL-1R1, TNF
C-C chemokine receptor type 5 (CCR5) signalling in macrophages	[19]	NS		0.0019	PLCG2, GNG2, CD3E, JUN, GNAI3, MAP2K4, CD3G, FAS, PRKCZ, CCL3, CCL5, GNG4, CCL4, GNB5
Triggering receptor expressed on myeloid cells 1 (TREM1) signalling	[20]	NS		0.0025	STAT5B, PLCG2, IL-18, NLRP6, NLRP3, TLR4, CCL3, NOD2, IL-1RL1, STAT3, NLRC4, TNF
Th1 pathway	[13]	NS		0.0032	IL-18, STAT4, PIK3CG, KL, SOCS3, TNFSF11, NFATC2, CD3E, IL-12B, IRS2, IL-27RA, IL-18R1, CD3G, NFIL-3, KLRD1, STAT3

Ingenuity canonical pathways	Association with COPD*	CHF6001 800 µg BID		CHF6001 1600 µg BID	
		p-value (FDR)†	Genes‡	p-value (FDR)*	Genes‡
Dendritic cell maturation	[21]	NS		0.0038	IL-1RL2, IL-18, STAT4, PIK3CG, IL-32, FSCN1, TLR4, KL, COL1A1, PLCG2, IL-12B, IRS2, MAP2K4, CD1B, LTB, IL-1A, FCGR2A, CD1A, PLCB1, TNF
Hepatocyte growth factor (HGF) signalling	[22]	NS		0.0038	PLCG2, RAPGEF1, IRS2, JUN, PIK3CG, MAP3K8, SOS2, MAP2K4, KL, PRKCZ, PAK1, STAT3, MAP3K2, ELF2, MAP3K13
Inflammasome pathway	[23]	NS		0.0038	IL-18, NOD2, NLRC4, NLRP3, P2RX7, TLR4
Role of pattern recognition receptors in recognition of bacteria and viruses	[24,25]	NS		0.0038	PLCG2, IL-12B, IRS2, IL-18, PIK3CG, MAP2K4, RNASEL, NLRP3, IL-1A, TLR4, KL, PRKCZ, NOD2, CCL5, NLRC4, TNF
Natural killer cell signalling	[26,27]	NS		0.0038	KIR2DL4, PLCG2, LCK, IRS2, SYNJ1, PIK3CG, INPP5D, SOS2, FCGR2A, KL, KLRD1, PRKCZ, KIR2DL2, PAK1, KLRB1

Ingenuity canonical pathways	Association with COPD*	CHF6001 800 µg BID		CHF6001 1600 µg BID	
		p-value (FDR)†	Genes‡	p-value (FDR)*	Genes‡
Molecular mechanisms of cancer		NS		0.0038	ARHGEF10, RAPGEF1, E2F7, PIK3CG, PRKAR1A, BCL2L11, SOS2, CDKN1B, AURKA, GAB2, BRAF, BMP6, IRS2, GNAI3, GNAT2, RALB, TFDP1, PRKCZ, PLCB1, JUN, FNBP1, KL, CDK16, PAK1, RHOF, CDK17, PRKDC, SMAD9, MAP2K4, CCNE2, RASA1, RHOU, FAS
Breast cancer regulation by stathmin1		NS		0.0041	PPP1CB, ARHGEF10, E2F7, PIK3CG, PRKAR1A, SOS2, CDKN1B, KL, PAK1, GNG4, PPP2R2A, GNB5, GNG2, TUBA4A, IRS2, CCNE2, GNAI3, PRKCZ, PLCB1, PPP2R5C, CAMK1
Granulocyte adhesion and diapedesis	[28]	NS		0.0049	IL-1RL2, MMP12, IL-18, IL-18RAP, FPR2, CCL5, MMP1, CCL4, FPR1, GNAI3, CXCR2, IL-1A, MMP7, CXCL6, CCL3, IL-1RL1, IL-1R1, TNF
Role of hypercytokinaemia/ hyperchemokinaemia in the pathogenesis of influenza		NS		0.0065	IL-12B, IL-18, IL-1A, CCR1, CCL3, CCL5, CCL4, TNF

Ingenuity canonical pathways	Association with COPD*	CHF6001 800 µg BID		CHF6001 1600 µg BID	
		p-value (FDR)†	Genes‡	p-value (FDR)*	Genes‡
Cholecystokinin/gastrin-mediated signalling		NS		0.0065	JUN, IL-18, FNBP1, SOS2, MAP2K4, IL-1A, RHOU, MEF2D, PRKCZ, RHOF, PLCB1, CREM, TNF
Differential regulation of cytokine production in intestinal epithelial cells by IL-17A and IL-17F		NS		0.0065	IL-12B, CCL3, CCL5, IL-1A, TNF, CCL4
Pathogenesis of multiple sclerosis		NS		0.0066	CCL3, CCL5, CCL4, CCR1
Osteoarthritis pathway		NS		0.0078	IL-1RL2, MMP12, IL-18RAP, HDAC4, SIK3, P2RX7, TLR4, ALPL, S1PR2, SMAD9, MMP1, MTF1, PRG4, CXCR2, DDIT4, TIMP3, IL-1RL1, PRKAB1, IL-1R1, TNF
High mobility group box 1 (HMGB1) signalling	[29]	NS		0.0083	IL-12B, IRS2, JUN, IL-18, PIK3CG, FNBP1, MAP2K4, IL-1A, RHOU, TLR4, KL, SERPINE1, RHOF, IL-1R1, TNF
Role of nuclear factor of activated T-cells (NFAT) in regulation of the immune response	[30]	NS		0.0083	LCK, JUN, PIK3CG, SOS2, KL, NFATC2, GNG4, GNB5, GNG2, PLCG2, CD3E, IRS2, GNAI3, GNAT2, FCGR2A, CD3G, MEF2D, PLCB1

Ingenuity canonical pathways	Association with COPD*	CHF6001 800 µg BID		CHF6001 1600 µg BID	
		p-value (FDR)†	Genes‡	p-value (FDR)*	Genes‡
Nuclear factor kappa light chain enhancer of activated B cells (NF-κB) signalling	[31]	NS		0.0083	LCK, IL-18, PELI1, PIK3CG, MAP3K8, TLR4, KL, TNFSF11, BRAF, PLCG2, IGF1R, IRS2, TNFSF13B, IL-1A, PRKCZ, MAP4K4, IL-1R1, TNF
Systemic lupus erythematosus signalling		NS		0.0087	LCK, JUN, IL-18, PIK3CG, CD22, SOS2, KL, NFATC2, CREM, PLCG2, CD3E, IRS2, INPP5D, TNFSF13B, IL-1A, FCGR2A, PTPRC, CD3G, TNF
T cell receptor signalling	[13]	NS		0.0093	LCK, CD3E, IRS2, JUN, PIK3CG, SOS2, MAP2K4, RASA1, CD3G, PTPRC, KL, BMX, NFATC2
Production of nitric oxide and reactive oxygen species in macrophages	[32]	NS		0.0098	PPP1CB, JUN, PIK3CG, FNBP1, MAP3K8, NCF1, TLR4, KL, RHOF, MAP3K2, PPP2R2A, PLCG2, IRS2, MAP2K4, RHOU, PRKCZ, PPP2R5C, MAP3K13, TNF
Renin-angiotensin signalling		NS		0.0098	PLCG2, IRS2, JUN, PIK3CG, PRKAR1A, SOS2, MAP2K4, KL, PRKCZ, PAK1, CCL5, STAT3, PTGER2, TNF

Ingenuity canonical pathways	Association with COPD*	CHF6001 800 µg BID		CHF6001 1600 µg BID	
		p-value (FDR)†	Genes‡	p-value (FDR)*	Genes‡
Phospholipase C signalling	[3]	NS		0.0098	PPP1CB, ARHGEF10, LCK, FNBP1, SOS2, HDAC4, PLD2, RHOF, NFATC2, GNG4, GNB5, GNG2, PLCG2, CD3E, FCGR2A, RALB, CD3G, RHOU, MEF2D, PRKCZ, PLCB1
Differential regulation of cytokine production in macrophages and t helper cells by IL-17A and IL-17F	[33,34]	NS		0.0098	IL-12B, CCL3, CCL5, TNF, CCL4
IL-8 signalling	[13]	NS		0.0098	DEFA1 (includes others), JUN, PIK3CG, FNBP1, KL, PLD2, RHOF, GNG4, BRAF, GNB5, GNG2, IRS2, MAP2K4, GNAI3, CXCR2, CXCR1, RHOU, PRKCZ, MAP4K4
Th2 pathway	[18]	NS		0.0098	STAT5B, IL-12B, CD3E, IRS2, JUN, STAT4, PIK3CG, CD3G, CRLF2, CCR1, KL, SOCS3, IL-4R, IL-1RL1, NFATC2
IL-3 signalling	[35]	NS		0.0098	STAT5B, RAPGEF1, IRS2, JUN, INPP5D, PIK3CG, KL, PRKCZ, PAK1, GAB2, STAT3

Ingenuity canonical pathways	Association with COPD*	CHF6001 800 µg BID		CHF6001 1600 µg BID	
		p-value (FDR)†	Genes‡	p-value (FDR)*	Genes‡
Prolactin signalling	[36]	NS		0.0098	STAT5B, PLCG2, IRS2, JUN, PIK3CG, SOS2, SOCS3, KL, PRL, PRKCZ, STAT3
Cardiac hypertrophy signalling		NS		0.0112	JUN, PIK3CG, PRKAR1A, FNBP1, MAP3K8, KL, RHOF, MAP3K2, GNG4, GNB5, GNG2, PLCG2, IRS2, IGF1R, MAP2K4, GNAI3, GNAT2, RHOU, MEF2D, PLCB1, MAP3K13
B cell receptor signalling	[35]	NS		0.0117	SYNJ1, JUN, PIK3CG, MAP3K8, CD22, EBF1, SOS2, KL, NFATC2, GAB2, MAP3K2, PLCG2, IRS2, INPP5D, MAP2K4, FCGR2A, PTPRC, MAP3K13
Stress-activated protein kinase/c-Jun NH2-terminal kinase (SAPK/JNK) signalling	[37]	NS		0.0117	GNG2, DUSP4, LCK, IRS2, JUN, PIK3CG, SOS2, MAP2K4, KL, MAP4K4, MAP3K2, MAP3K13
Ephrin receptor signalling		NS		0.0135	RAPGEF1, PIK3CG, SOS2, PAK1, GNG4, GNB5, GNG2, PDGFB, PDGFA, GNAI3, GNAT2, RASA1, EFNA1, ITSN1, MAP4K4, STAT3, EFNB2

Ingenuity canonical pathways	Association with COPD*	CHF6001 800 µg BID		CHF6001 1600 µg BID	
		p-value (FDR)[†]	Genes[‡]	p-value (FDR)*	Genes[‡]
Thrombopoietin signalling	[38]	NS		0.0170	STAT5B, PLCG2, IRS2, JUN, PIK3CG, KL, PRKCZ, GAB2, STAT3
G Protein signalling mediated by tubby		NS		0.0170	GNG2, PLCG2, LCK, PLCB1, GNG4, GNB5
Altered T cell and B cell signalling in rheumatoid arthritis		NS		0.0170	IL-12B, CSF1, IL-18, TNFSF13B, LTB, IL-1A, TLR4, TNFSF11, FAS, TNF
Colorectal cancer metastasis signalling		NS		0.0170	MMP12, JUN, PIK3CG, PRKAR1A, FNBP1, SOS2, TLR4, KL, RHOF, GNG4, BRAF, MMP1, GNB5, GNG2, IRS2, MAP2K4, MMP7, RHOU, STAT3, PTGER2, TNF
C-X-C chemokine receptor type 4 (CXCR4) signalling	[39]	NS		0.0174	JUN, PIK3CG, FNBP1, KL, RHOF, PAK1, GNG4, GNB5, GNG2, IRS2, MAP2K4, GNAI3, GNAT2, RHOU, PRKCZ, PLCB1
Phagosome formation	[40]	NS		0.0186	PLCG2, IRS2, PIK3CG, INPP5D, FNBP1, FCGR2A, RHOU, TLR4, KL, PRKCZ, RHOF, PLCB1, FCAR

Ingenuity canonical pathways	Association with COPD*	CHF6001 800 µg BID		CHF6001 1600 µg BID	
		p-value (FDR)†	Genes‡	p-value (FDR)*	Genes‡
Role of JAK2 in hormone-like cytokine signalling	[17]	NS		0.0186	STAT5B, SOCS3, PRL, IRS2, STAT3, EPOR
N-Formylmethionyl-leucyl-phenylalanine (fMLP) signalling in neutrophils	[41]	NS		0.0195	GNG2, FPR1, IRS2, PIK3CG, GNAI3, FPR2, NCF1, KL, PRKCZ, PLCB1, NFATC2, GNG4, GNB5
Erythropoietin signalling	[42]	NS		0.0200	STAT5B, PLCG2, IRS2, JUN, PIK3CG, SOS2, EPOR, SOCS3, KL, PRKCZ
Chemokine signalling	[13]	NS		0.0200	PPP1CB, PLCG2, JUN, PIK3CG, GNAI3, CCL5, PLCB1, CCL4, CAMK1
Role of JAK1 and JAK3 in γc cytokine signalling	[17]	NS		0.0214	STAT5B, IRS2, PIK3CG, FES, CRLF2, SOCS3, IL-4R, KL, STAT3
Cardiac β-adrenergic signalling		NS		0.0219	ATP2A3, PPP1CB, GNG2, PDE4D, PDE4B, PRKAR1A, PDE7A, AKAP12, PDE2A, PPP2R5C, AKAP13, GNG4, PPP2R2A, GNB5
IL-9 signalling	[43]	NS		0.0219	STAT5B, KL, SOCS3, IRS2, STAT3, PIK3CG, TNF

Ingenuity canonical pathways	Association with COPD*	CHF6001 800 μg BID		CHF6001 1600 μg BID	
		p-value (FDR)†	Genes‡	p-value (FDR)*	Genes‡
fms like tyrosine kinase 3 (FLT3) signalling in hematopoietic progenitor cells		NS		0.0219	STAT5B, IRS2, STAT4, INPP5D, PIK3CG, SOS2, KL, GAB2, FLT3, STAT3
ErbB signalling	[44]	NS		0.0224	PLCG2, EREG, IRS2, JUN, PIK3CG, SOS2, MAP2K4, KL, PRKCZ, AREG, PAK1
IL-22 signalling	[13]	NS		0.0229	STAT5B, IL-22RA2, SOCS3, STAT3, MAP2K4
Growth hormone signalling	[45]	NS		0.0229	STAT5B, PLCG2, IRS2, IGF1R, PIK3CG, SOCS3, KL, PRL, PRKCZ, STAT3
Glioma signalling		NS		0.0240	PDGFB, PLCG2, PDGFA, IRS2, IGF1R, E2F7, PIK3CG, SOS2, TFDP1, KL, PRKCZ, CAMK1
Crosstalk between dendritic cells and natural killer cells	[21,26,27]	NS		0.0240	KIR2DL4, IL-12B, IL-18, LTB, FSCN1, TLR4, KLRD1, FAS, KIR2DL2, TNF
Gαq signalling	[46]	NS		0.0245	PLCG2, GNG2, IRS2, PIK3CG, FNBP1, RHOU, PLD2, KL, PRKCZ, RHOF, NFATC2, PLCB1, GNG4, RGS18, GNB5

Ingenuity canonical pathways	Association with COPD*	CHF6001 800 µg BID		CHF6001 1600 µg BID	
		p-value (FDR)†	Genes‡	p-value (FDR)*	Genes‡
Toll-like receptor signalling	[47]	NS		0.0288	IL-12B, JUN, IL-18, MAP2K4, IL-1A, TLR4, IL-1RL1, MAP4K4, TNF
Cell cycle regulation by BTG family proteins	[48]	NS		0.0302	NOCT, E2F7, PPP2R5C, BTG1, CCNE2, PPP2R2A
Protein kinase c theta (PKCθ) signalling in T lymphocytes	[49]	NS		0.0324	PLCG2, LCK, CD3E, IRS2, JUN, PIK3CG, MAP3K8, SOS2, MAP2K4, CD3G, KL, NFATC2, MAP3K2, MAP3K13
Glial cell-derived neurotrophic factor (GDNF) family ligand-receptor interactions		NS		0.0331	PLCG2, IRS2, JUN, PIK3CG, SOS2, MAP2K4, RASA1, DOK5, KL
Acute myeloid leukaemia signalling		NS		0.0331	STAT5B, IRS2, PIK3CG, SOS2, MAP2K4, RARA, KL, FLT3, STAT3, BRAF
Glutathione biosynthesis	[50]	NS		0.0331	GCLM, GCLC
Insulin-like growth factor (IGF)-1 signalling	[45]	NS		0.0339	IRS2, IGF1R, JUN, PIK3CG, PRKAR1A, SOS2, RASA1, SOCS3, KL, PRKCZ, STAT3
Atherosclerosis signalling		NS		0.0347	PDGFB, PDGFA, CSF1, IL-18, PLB1, IL-1A, PNPLA8, COL1A1, ALOX15B, CD36, MMP1, TNF

Ingenuity canonical pathways	Association with COPD*	CHF6001 800 μg BID		CHF6001 1600 μg BID	
		p-value (FDR)†	Genes‡	p-value (FDR)*	Genes‡
PPARα/RXRα activation	[14]	NS		0.0347	STAT5B, PLCG2, IL-1RL2, JUN, IL-18RAP, PRKAR1A, SOS2, MAP2K4, GK, IL-1RL1, MAP4K4, PLCB1, PRKAB1, CD36, IL-1R1
Ceramide signalling	[51]	NS		0.0347	IRS2, JUN, PIK3CG, MAP2K4, KL, PRKCZ, S1PR2, PPP2R5C, TNF, PPP2R2A
IL-1 signalling	[13]	NS		0.0347	GNG2, JUN, PRKAR1A, GNAI3, MAP2K4, GNAT2, IL-1A, IL-1R1, GNG4, GNB5
Communication between innate and adaptive immune cells	[52]	NS		0.0347	IL-12B, IL-18, TNFSF13B, IL-1A, TLR4, CCL3, CCL5, CCL4, TNF
Thrombin signalling		NS		0.0347	PPP1CB, ARHGEF10, PIK3CG, FNBP1, KL, RHOF, GNG4, GNB5, GNG2, PLCG2, IRS2, GNAI3, GNAT2, RHOU, PRKCZ, PLCB1, CAMK1
Sphingosine-1-phosphate signalling	[51]	NS		0.0347	PDGFB, PLCG2, PDGFA, IRS2, PIK3CG, FNBP1, GNAI3, RHOU, KL, RHOF, PLCB1, S1PR2

Ingenuity canonical pathways	Association with COPD*	CHF6001 800 µg BID		CHF6001 1600 µg BID	
		p-value (FDR)†	Genes‡	p-value (FDR)*	Genes‡
Phosphoinositide 3-kinase/protein kinase B (PI3K/AKT) signalling	[9]	NS		0.0347	GDF15, MCL1, SYNJ1, INPP5D, PIK3CG, MAP3K8, SOS2, CDKN1B, PRKCZ, GAB2, PPP2R5C, PPP2R2A
Agranulocyte adhesion and diapedesis	[28]	NS		0.0398	MMP12, IL-18, CXCR2, GNAI3, CXCL6, MMP7, IL-1A, MYH10, CXCR1, CCL3, CCL5, IL-1R1, MMP1, TNF, CCL4
Protein kinase A signalling	[11,12]	NS		0.0427	PPP1CB, DUSP4, PDE4D, PDE4B, PRKAR1A, DUSP6, MYH10, AKAP12, NFATC2, AKAP13, EBI3, CREM, GNG4, BRAF, GNB5, PLCG2, GNG2, GNAI3, MYLK3, MTMR3, DUSP16, PTPRC, PTP4A1, PRKCZ, PDE7A, PDE2A, PLCB1
ErbB2-ErbB3 signalling	[44]	NS		0.0437	STAT5B, IRS2, JUN, PIK3CG, SOS2, CDKN1B, KL, STAT3
Epidermal growth factor (EGF) signalling	[53]	NS		0.0437	KL, IRS2, JUN, STAT3, PIK3CG, SOS2, MAP2K4, RASA1
Calcium-induced T lymphocyte apoptosis	[54]	NS		0.0457	ATP2A3, MEF2D, PRKCZ, CD3E, LCK, NFATC2, CD3G

Ingenuity canonical pathways	Association with COPD*	CHF6001 800 µg BID		CHF6001 1600 µg BID	
		p-value (FDR)†	Genes‡	p-value (FDR)*	Genes‡
JAK/signal transducers and activators of transcription (Stat) signalling	[17]	NS		0.0457	STAT5B, IRS2, JUN, STAT4, PIK3CG, SOS2, SOCS3, KL, STAT3
Glioblastoma multiforme signalling		NS		0.0457	PLCG2, PDGFB, PDGFA, IRS2, IGF1R, E2F7, PIK3CG, FNBP1, SOS2, CDKN1B, RHOU, KL, RHOF, PLCB1
G beta gamma signalling	[10]	NS		0.0490	GNG2, PLCG2, PIK3CG, PRKAR1A, SOS2, GNAI3, GNAT2, PRKCZ, PAK1, GNG4, GNB5

Abbreviations: BID, twice daily; NS, not significant; IL, interleukin; Th, T helper; JAK, Janus kinase; PPAR, peroxisome proliferator-activated receptor; RXR, retinoid X receptor.

Figure S3. Graphical gene relationship simulation of perturbations using Ingenuity Pathway Analysis Molecule Activity Predictor (MAP) for CHF6001 1600 µg COPD pathways associated with clear-cut and consistent inhibition of downstream inflammatory mediators or conditions

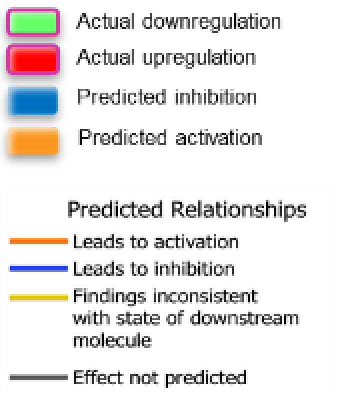

C-C chemokine receptor type 5 (CCR5) signalling in macrophages

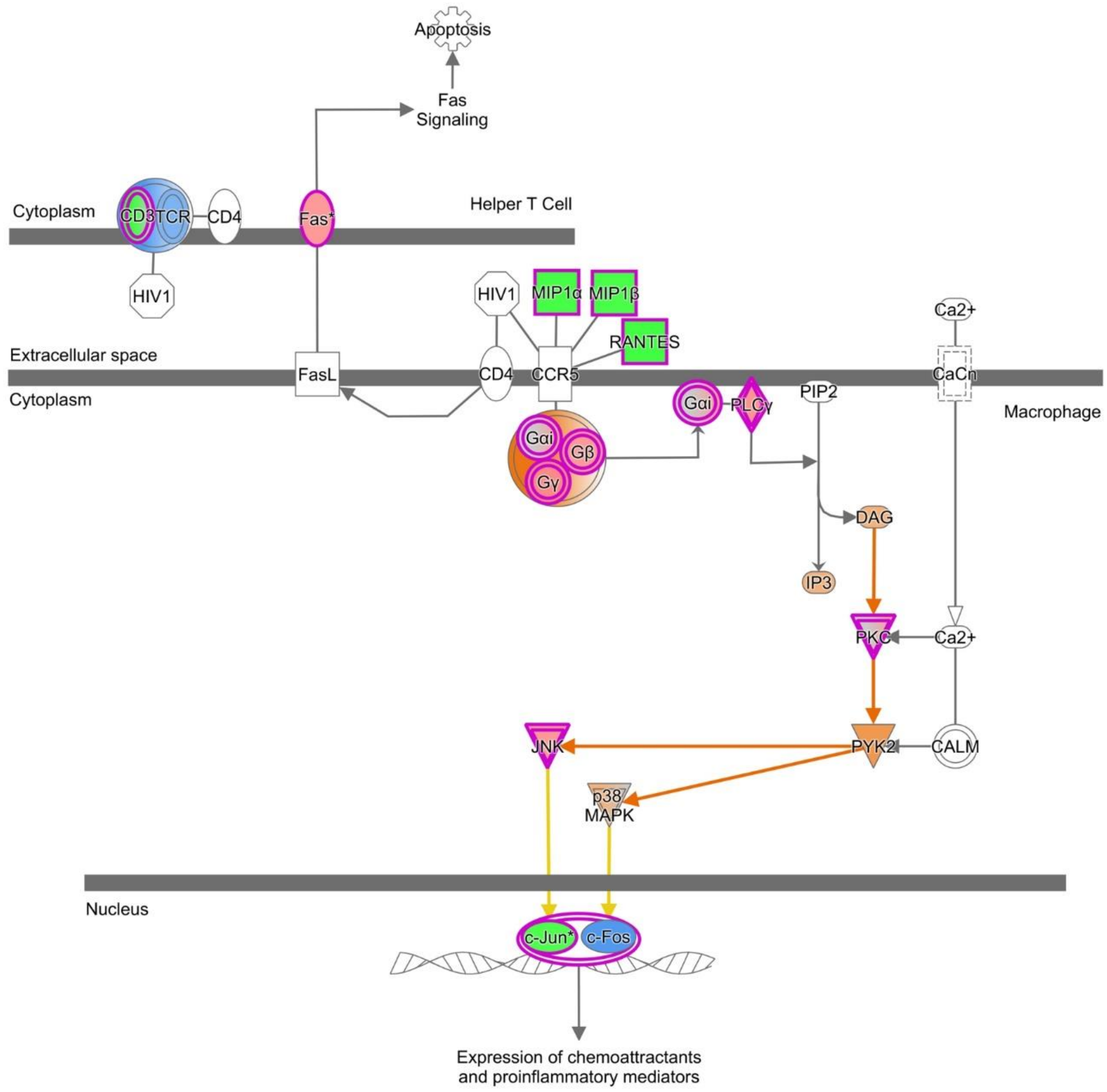

Chemokine signalling

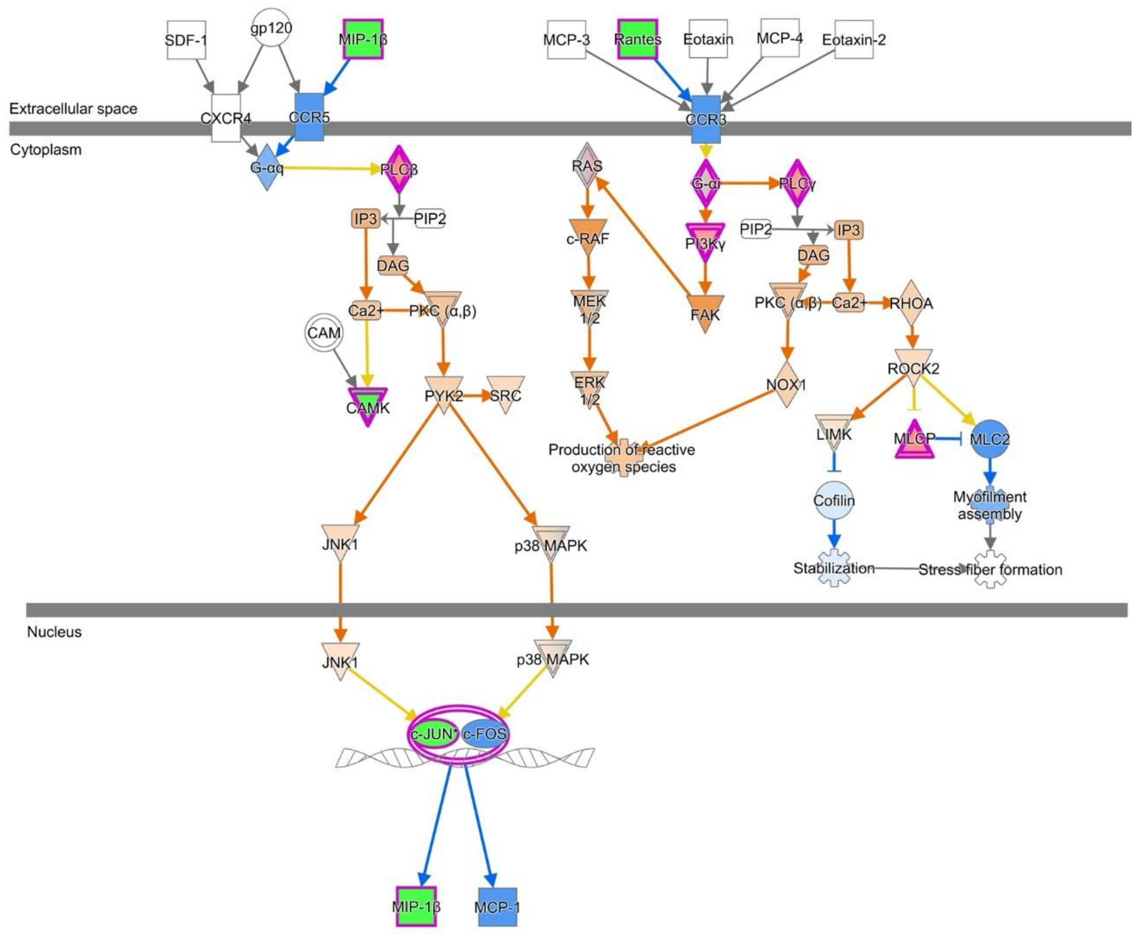

High mobility group-B1 (HMGB1) signalling

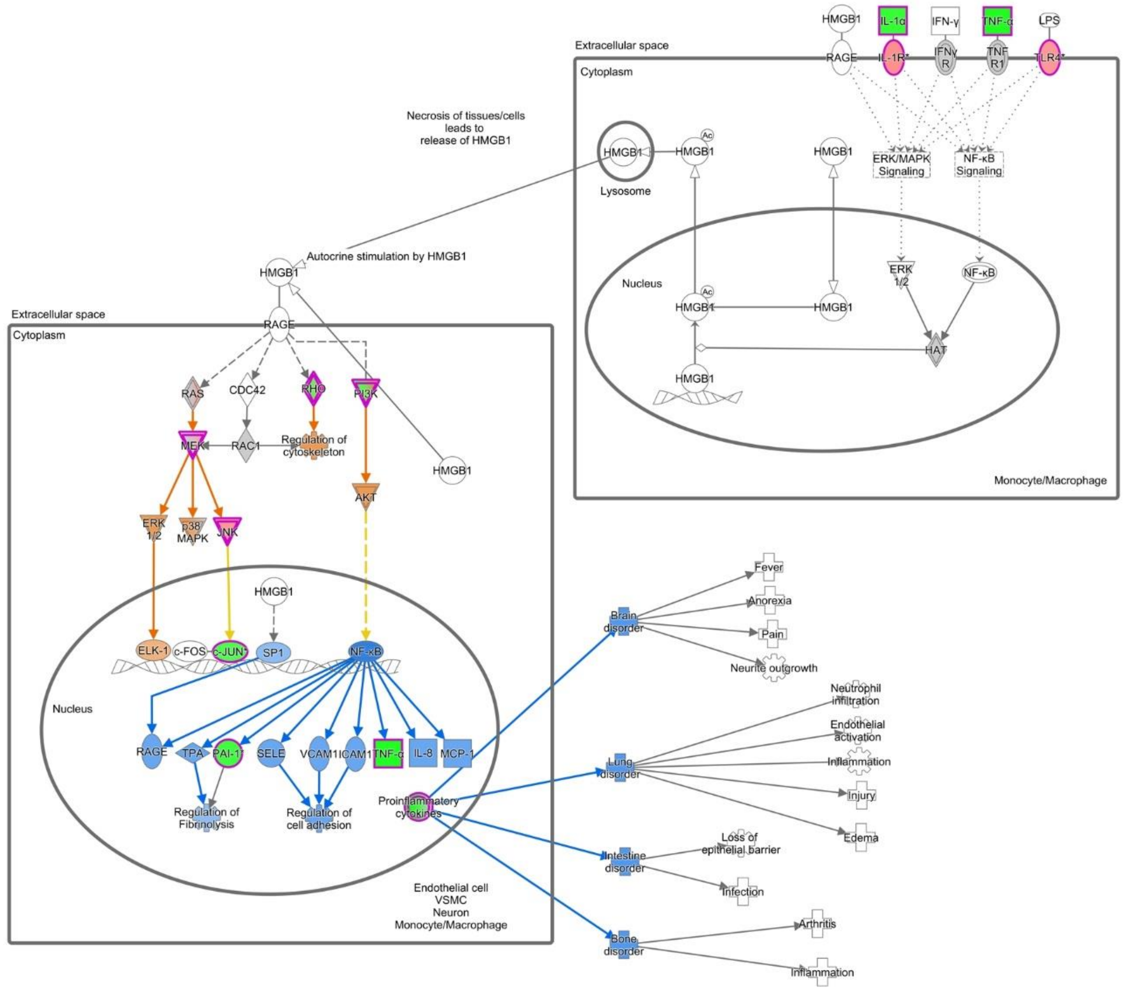

Triggering receptor expressed on myeloid cells 1 (TREM1) signalling

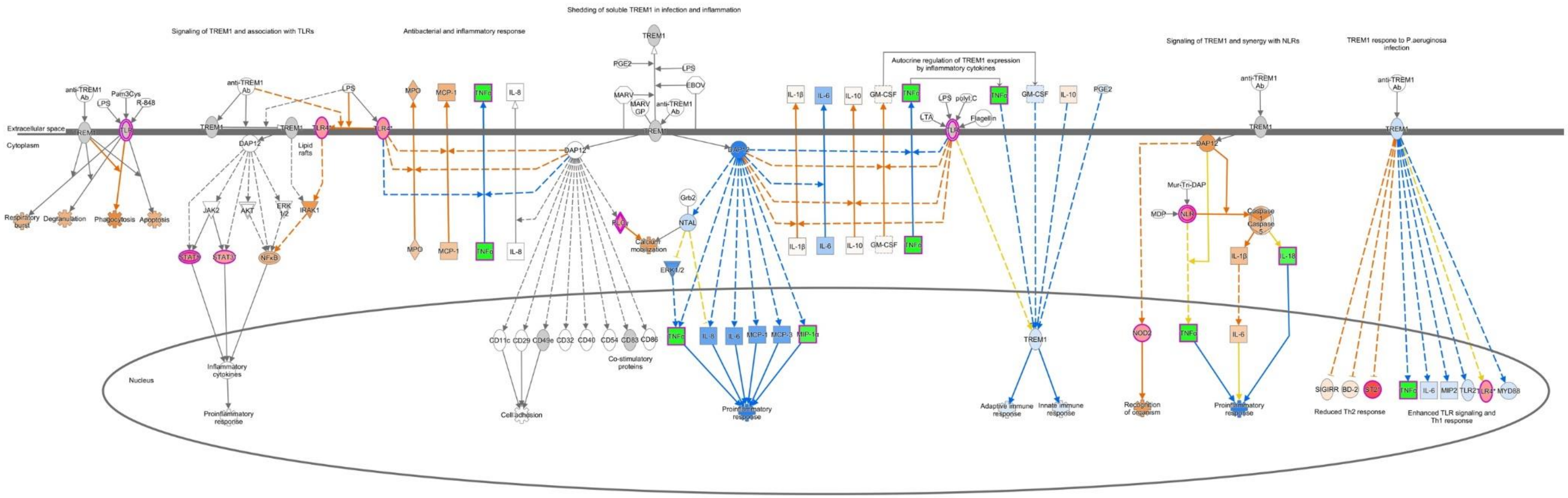

Interleukin 22 signalling

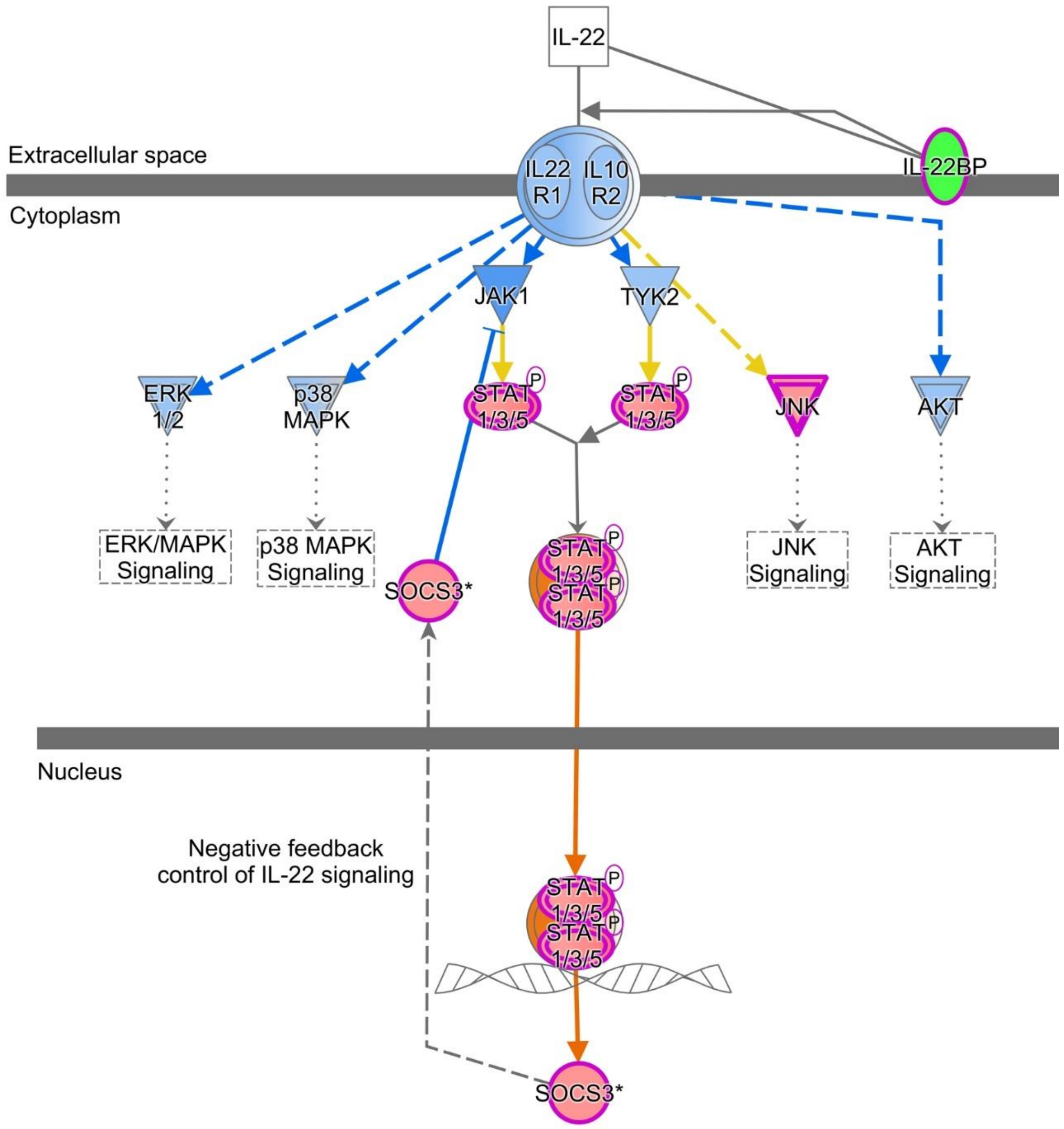

Ceramide signalling

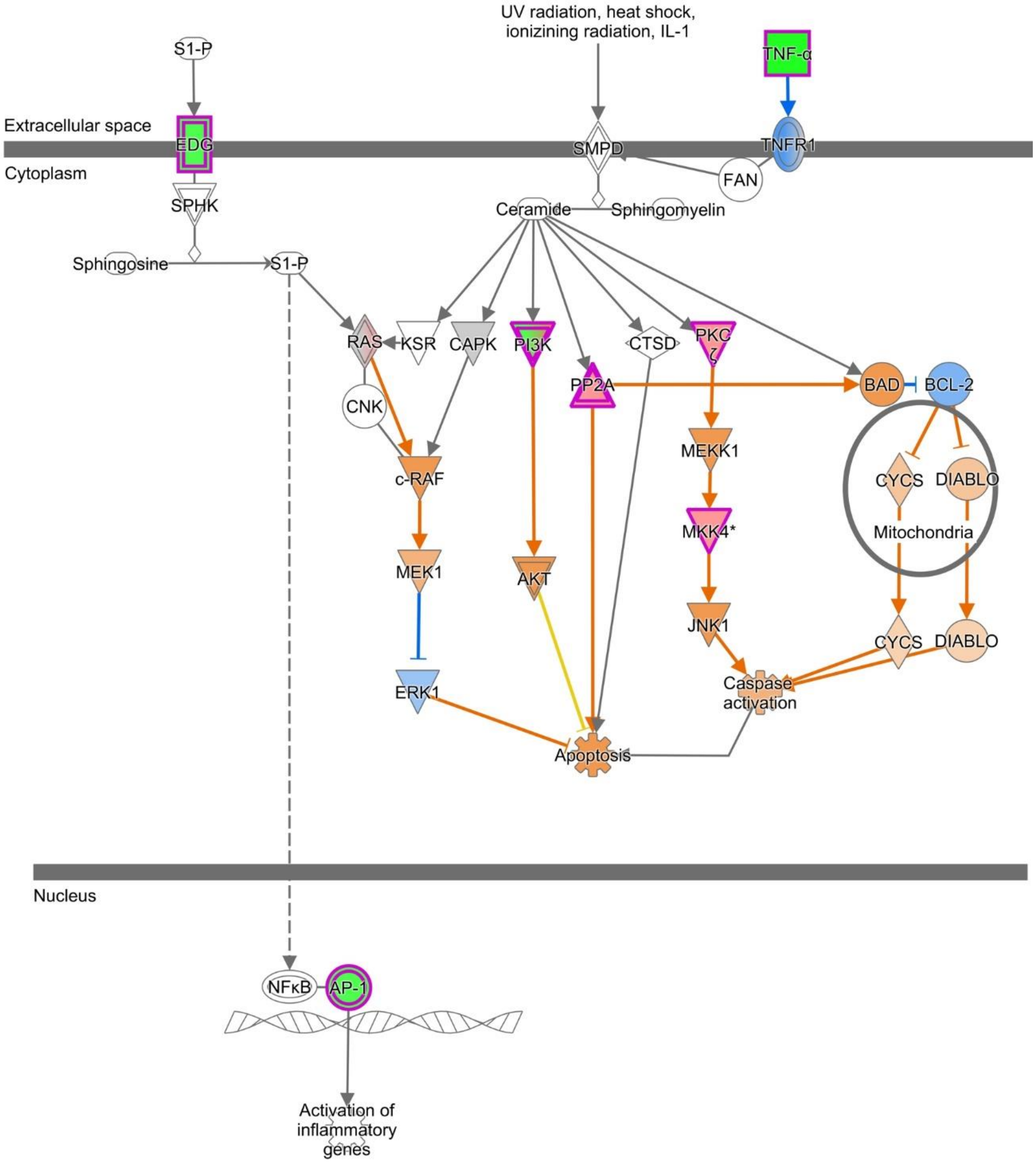

Protein kinase c theta (PKCθ) signalling in T lymphocytes

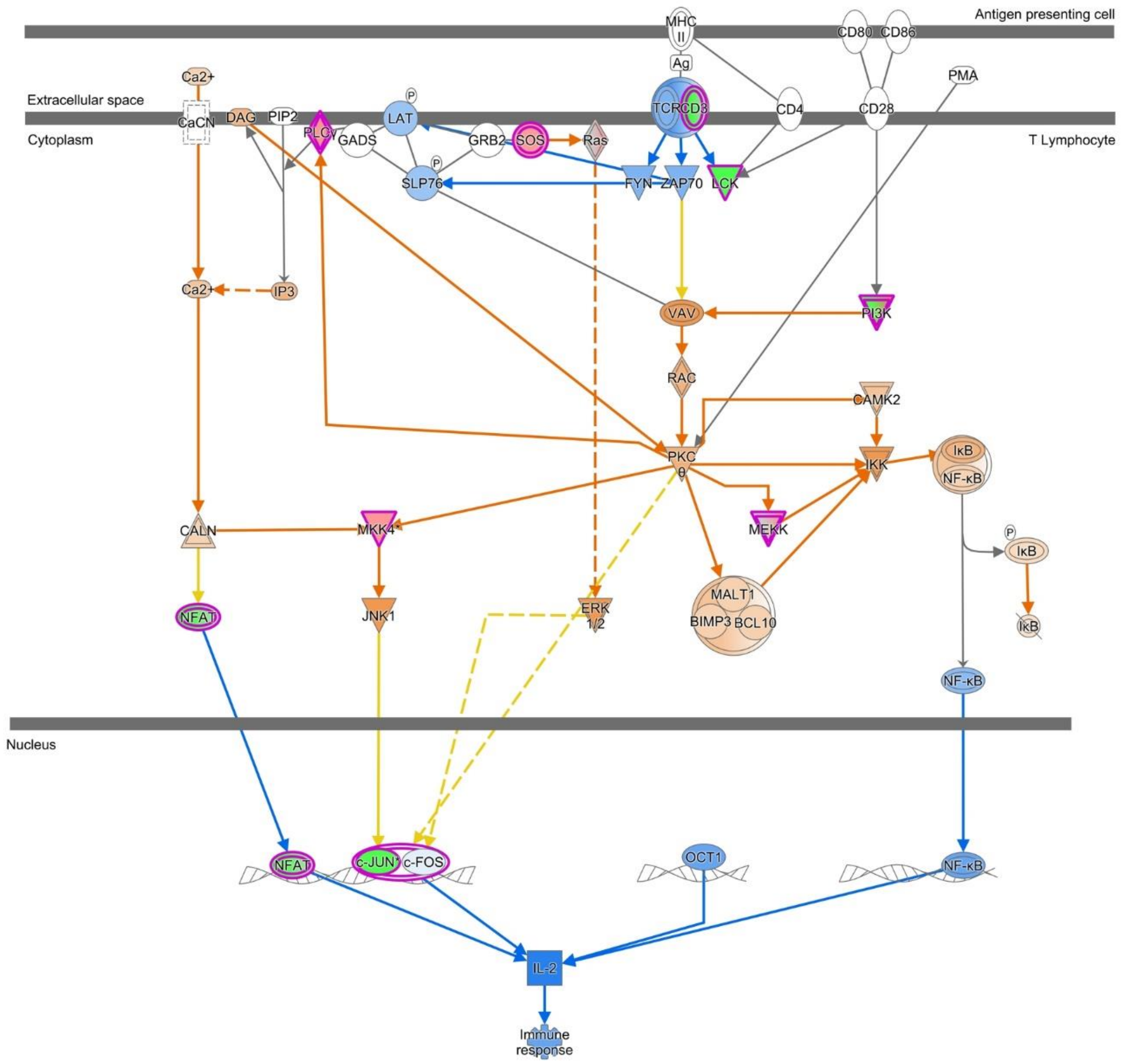

Role of pattern recognition receptors in recognition of bacteria and viruses

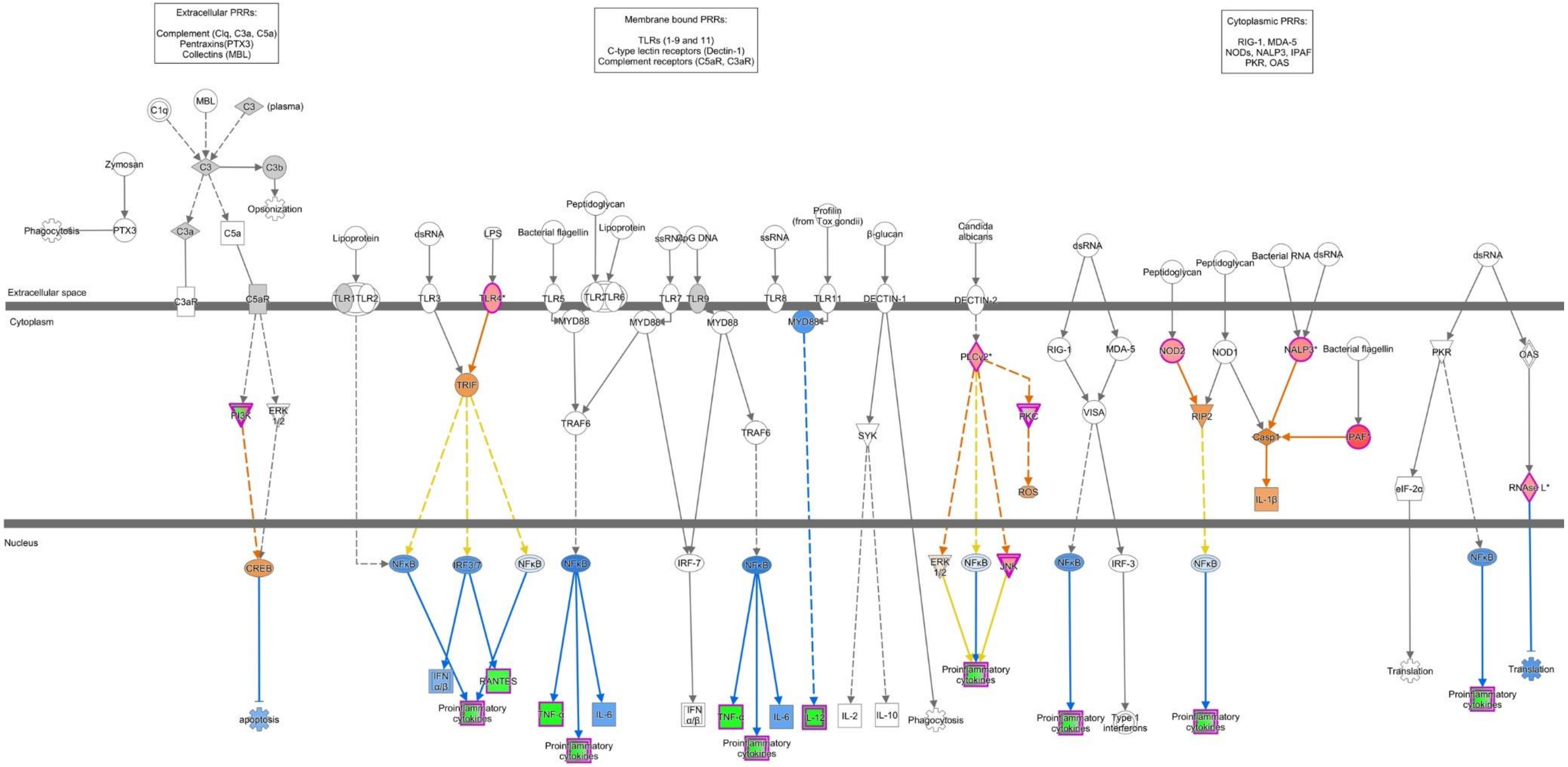

Toll-like receptors signalling

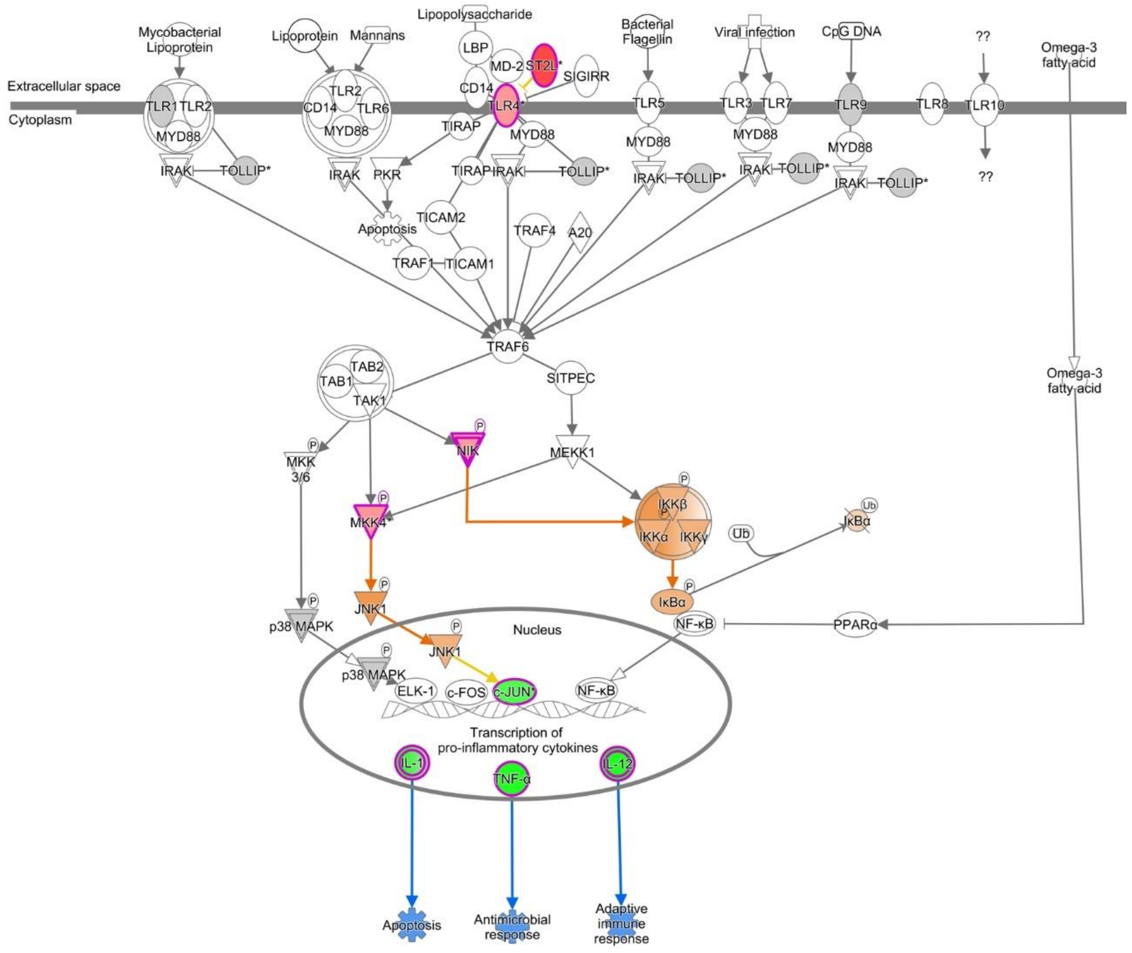

N-formylmethionyl-leucyl-phenylalanine (fMLP) signalling in neutrophils

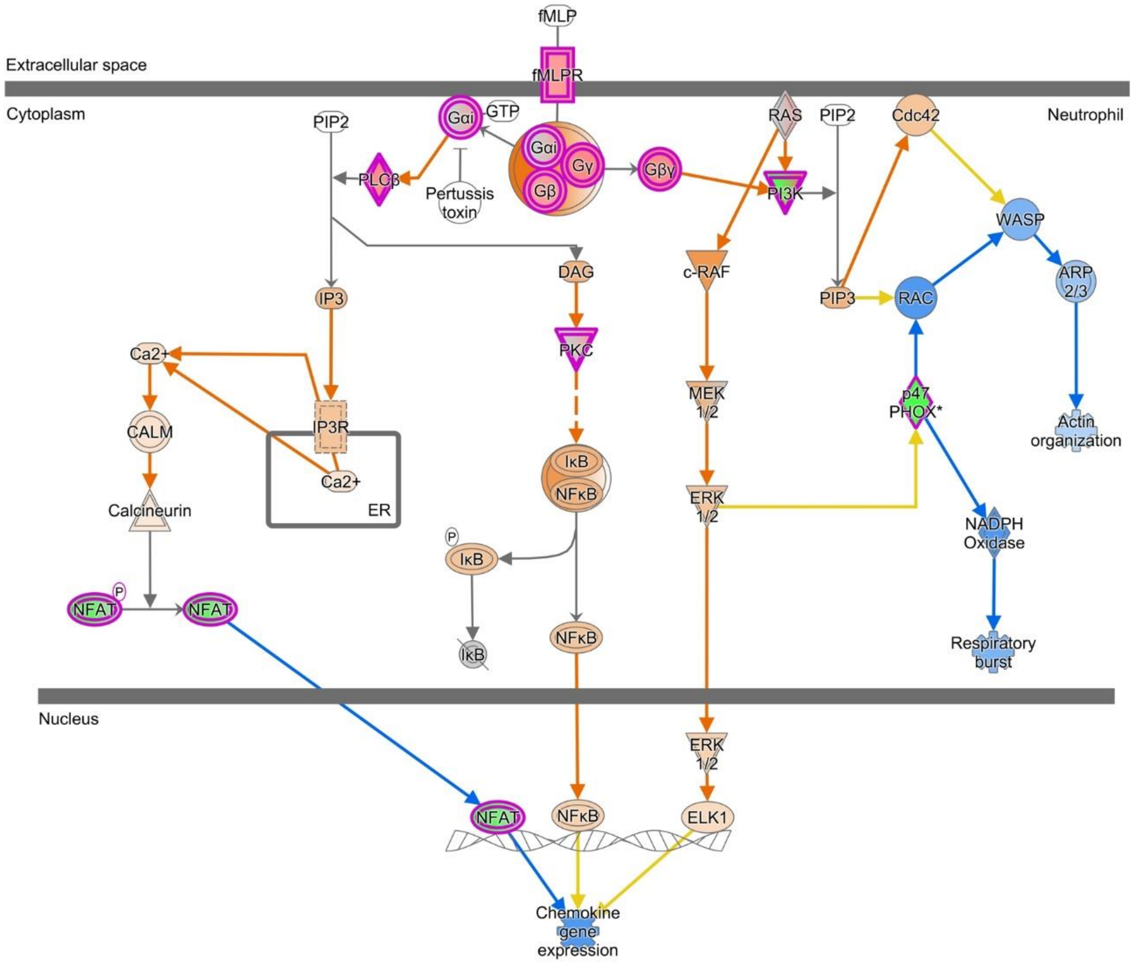

Production of nitric oxide and reactive oxygen species in macrophages

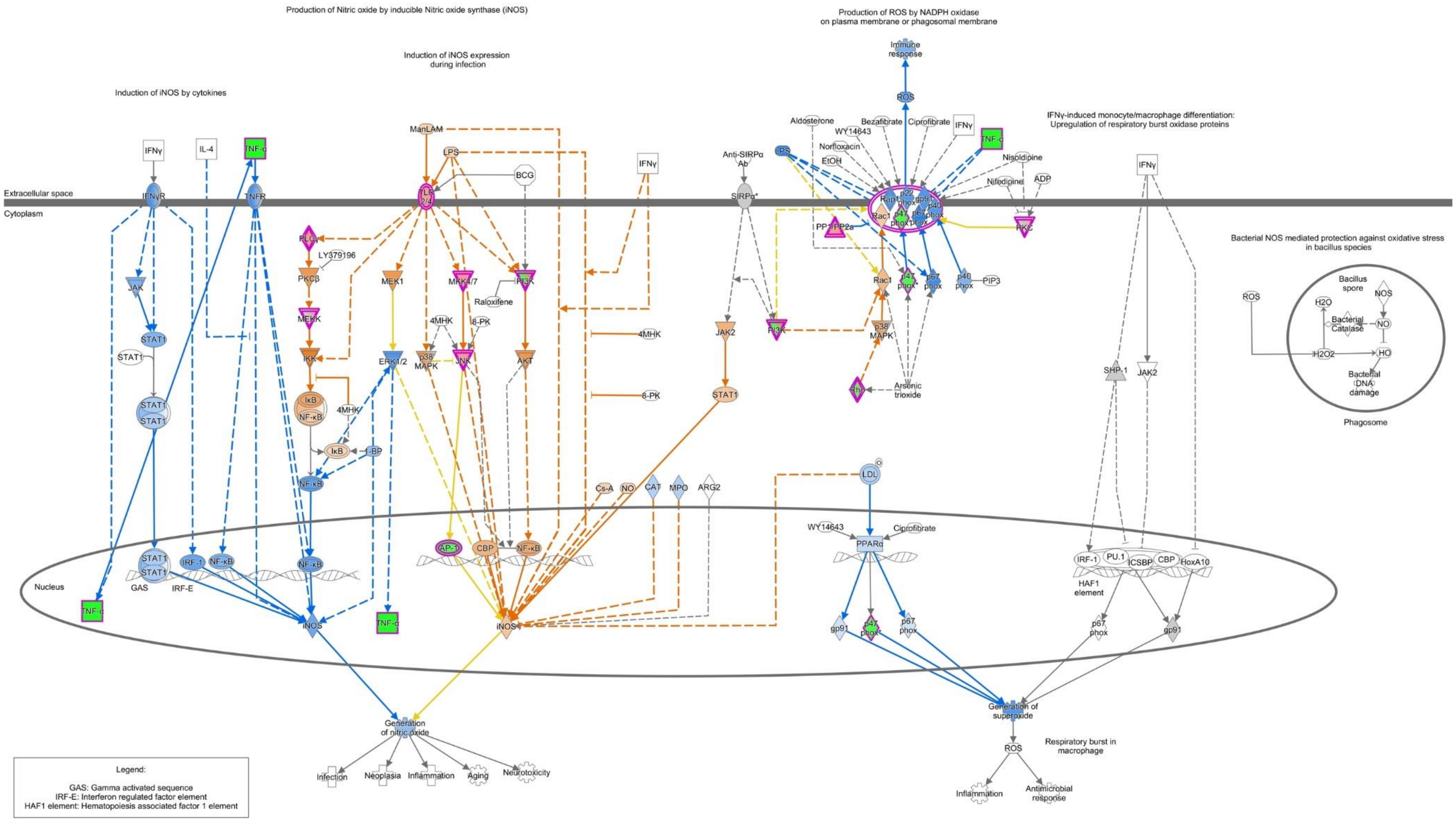

Vitamin D receptor activation

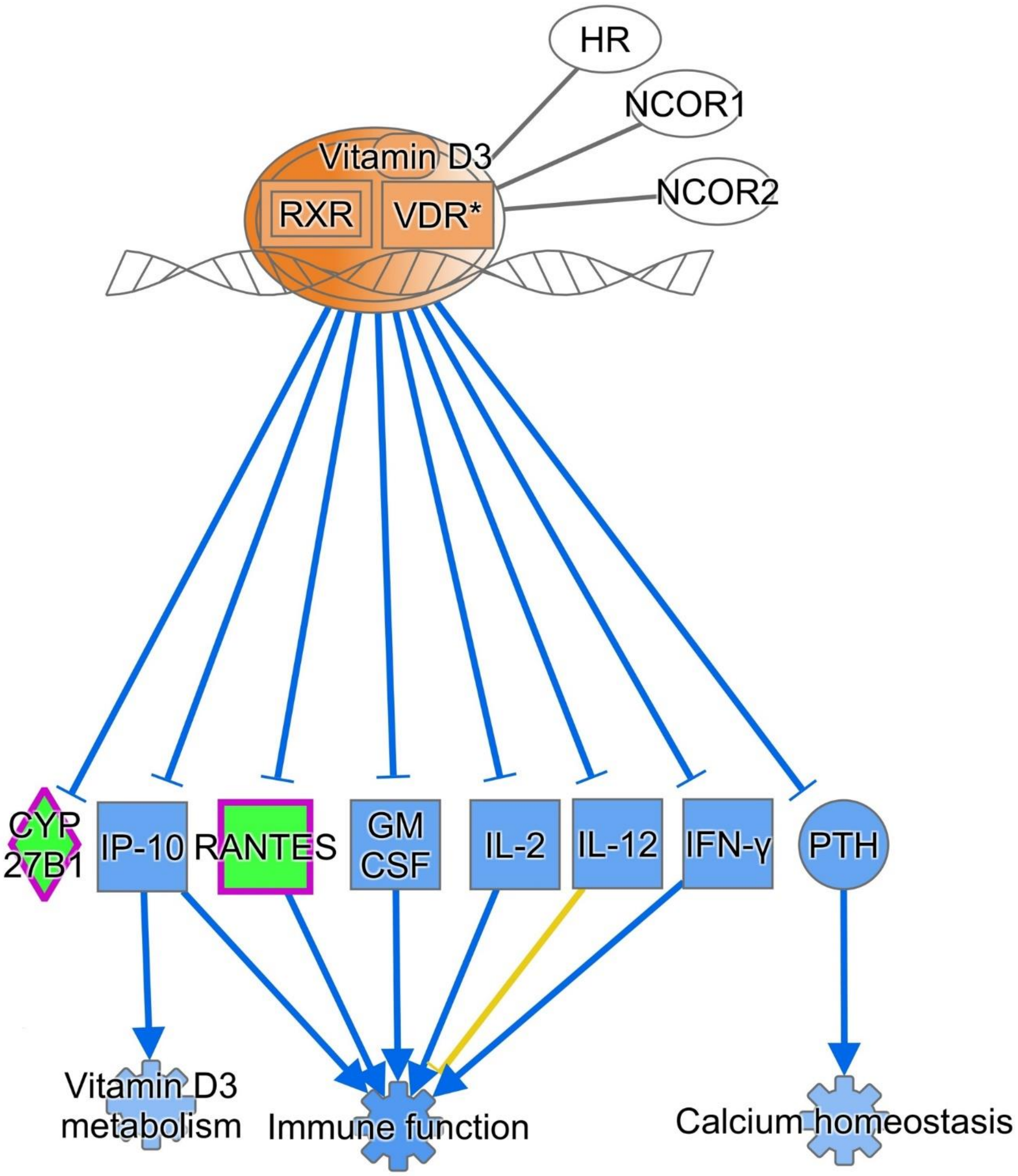

Table S2. Top 10 gene ontology biological processes and gene ontology molecular functions significantly affected by CHF6001.

	**CHF6001 800 µg BID**		**CHF6001 1600 µg BID**	
	**Description**	**pFDR**	**Description**	**pFDR**
Biological process	programmed cell death	<0.0001	immune system process	<0.0001
	regulation of apoptotic process	<0.0001	regulation of response to stimulus	<0.0001
	apoptotic process	<0.0001	immune response	<0.0001
	regulation of programmed cell death	<0.0001	intracellular signal transduction	<0.0001
	cell death	<0.0001	regulation of intracellular signal transduction	<0.0001
	signal transduction	0.0001	leukocyte activation	<0.0001
	immune system process	0.0001	signal transduction	<0.0001
	positive regulation of biological process	0.0001	cell activation	<0.0001
	regulation of cell death	0.0001	positive regulation of biological process	<0.0001
	regulation of response to stimulus	0.0002	single organism signalling	<0.0001

	CHF6001 800 µg BID		CHF6001 1600 µg BID	
	**Description**	**pFDR**	**Description**	**pFDR**
Molecular function	cytokine receptor activity	0.0042	cytokine receptor activity	0.0058
	interleukin-1 receptor activity	0.0042	ribonucleotide binding	0.0103
	pantetheine hydrolase activity	0.0042	cytokine activity	0.0103
	phosphotransferase activity, alcohol group as acceptor	0.0323	purine ribonucleotide binding	0.0103
	interleukin-1 binding	0.0471	purine nucleotide binding	0.0103
	3',5'-cyclic-AMP phosphodiesterase activity	0.0471	purine ribonucleoside binding	0.0103
	kinase activity	0.0471	pantetheine hydrolase activity	0.0103
	transferase activity, transferring phosphorus-containing groups	0.0471	purine nucleoside binding	0.0103
	interleukin-1, Type I, activating receptor activity	0.0471	ribonucleoside binding	0.0103
	interleukin-8 receptor activity	0.0471	carbohydrate derivative binding	0.0103

Abbreviations: BID, twice daily.

Inflammatory gene interaction network analysis

Table S3. Proinflammatory cytokines and matrix-metalloproteinases differentially expressed (pFDR<0.05 or p<0.05 and |FC|>1.3) after treatment with CHF6001 800 µg BID relative to placebo. ¥ common genes with CHF6001 1600 µg BID.

Probe set_ID	Gene Symbol	Entrez Gene	Fold change vs. placebo	p value	pFDR value	Gene Title
**Cytokines**						
216598_s_at	CCL2	6347	–1.30	1.08E–03	3.70E–02	chemokine (C-C motif) ligand 2
205114_s_at	CCL3 /// CCL3L1 /// CCL3L3¥	6348 /// 6349 /// 414062	–1.27	3.60E–05	2.89E–03	chemokine (C-C motif) ligand 3 /// chemokine (C-C motif) ligand 3-like 1 /// chemokine (C-C motif) ligand 3-like 3
204103_at	CCL4¥	6351	–1.28	2.85E–05	2.38E–03	chemokine (C-C motif) ligand 4
1555759_a_at	CCL5¥	6352	–1.43	2.12E–03	5.90E–02	chemokine (C-C motif) ligand 5
208075_s_at	CCL7	6354	–1.32	2.56E–02	2.41E–01	chemokine (C-C motif) ligand 7
206336_at	CXCL6¥	6372	1.35	8.34E–03	1.35E–01	chemokine (C-X-C motif) ligand 6
203915_at	CXCL9	4283	–1.62	9.28E–03	1.43E–01	chemokine (C-X-C motif) ligand 9
204533_at	CXCL10	3627	–1.80	2.06E–02	2.16E–01	chemokine (C-X-C motif) ligand 10
210390_s_at	CCL15 /// CCL15-CCL14¥	6359 /// 348249	–1.81	3.78E–04	1.71E–02	chemokine (C-C motif) ligand 15 /// CCL15-CCL14 readthrough (NMD candidate)

Probe set_ID	Gene Symbol	Entrez Gene	Fold change vs. placebo	p value	pFDR value	Gene Title
219424_at	EBI3¥	10148	–1.42	5.08E–05	3.82E–03	Epstein-Barr virus induced 3
207901_at	IL-12B¥	3593	–2.23	4.37E–06	5.09E–04	interleukin 12B
205992_s_at	IL-15	3600	–1.32	5.14E–03	1.01E–01	interleukin 15
203828_s_at	IL-32¥	9235	–1.46	4.22E–04	1.86E–02	interleukin 32
1568574_x_at	SPP1	6696	–1.27	6.89E–04	2.70E–02	secreted phosphoprotein 1
207113_s_at	TNF¥	7124	–1.37	1.78E–02	2.01E–01	tumour necrosis factor
229814_at	TNFAIP8¥	25816	–1.33	4.18E–05	3.23E–03	tumour necrosis factor, alpha-induced protein 8
227420_at	TNFAIP8L1¥	126282	–1.35	8.20E–05	5.49E–03	tumour necrosis factor, alpha-induced protein 8-like 1
241819_at	TNFSF8¥	944	–1.33	6.75E–03	1.19E–01	tumour necrosis factor (ligand) superfamily, member 8
223502_s_at	TNFSF13B¥	10673	1.35	5.27E–06	5.90E–04	tumour necrosis factor (ligand) superfamily, member 13b
229242_at	TNFSF15¥	9966	–1.62	1.79E–04	9.88E–03	tumour necrosis factor (ligand) superfamily, member 15
**Matrix metalloproteinases**						
201069_at	MMP2¥	4313	–1.32	1.28E–02	1.70E–01	matrix metallopeptidase 2
204259_at	MMP7¥	4316	–1.71	5.44E–05	4.02E–03	matrix metallopeptidase 7
204580_at	MMP12¥	4321	–1.36	1.05E–04	6.64E–03	matrix metallopeptidase 12

Probe set_ID	Gene Symbol	Entrez Gene	Fold change vs. placebo	p value	pFDR value	Gene Title
202827_s_at	MMP14[¥]	4323	–1.30	6.09E–04	2.46E–02	Matrix metallopeptidase 14 (membrane-inserted)

Table S4. Proinflammatory cytokines and matrix-metalloproteinases differentially expressed (pFDR<0.05 or p<0.05 and |FC|>1.3) after treatment with CHF6001 1600 µg BID relative to placebo.¥ common genes with CHF6001 800 µg BID.

Probe set_ID	Gene Symbol	Entrez Gene	Fold change vs. placebo	p value	pFDR value	Gene Title
**Cytokines**						
205114_s_at	CCL3 /// CCL3L1 /// CCL3L3¥	6348 /// 6349 /// 414062	–1.39	3.67E–08	3.63E–06	chemokine (C-C motif) ligand 3 /// chemokine (C-C motif) ligand 3-like 1 /// chemokine (C-C motif) ligand 3-like 3
204103_at	CCL4¥	6351	–1.38	1.63E–07	1.34E–05	chemokine (C-C motif) ligand 4
1555759_a_at	CCL5¥	6352	–1.42	2.10E–03	3.13E–02	chemokine (C-C motif) ligand 5
210390_s_at	CCL15 /// CCL15-CCL14¥	6359 /// 348249	–1.50	1.16E–02	1.03E–01	chemokine (C-C motif) ligand 15 /// CCL15-CCL14 readthrough (NMD candidate)
209716_at	CSF1	1435	–1.35	1.53E–03	2.50E–02	colony stimulating factor 1 (macrophage)
206336_at	CXCL6¥	6372	1.44	1.60E–03	2.58E–02	chemokine (C-X-C motif) ligand 6
219424_at	EBI3¥	10148	–1.35	3.46E–04	8.00E–03	Epstein-Barr virus induced 3
210354_at	IFNG	3458	–1.52	1.85E–02	1.39E–01	interferon, gamma
210118_s_at	IL-1A	3552	–1.53	1.51E–03	2.48E–02	interleukin 1 alpha
207901_at	IL-12B¥	3593	–1.86	2.83E–04	6.84E–03	interleukin 12B

Probe set_ID	Gene Symbol	Entrez Gene	Fold change vs. placebo	p value	pFDR value	Gene Title
206295_at	IL-18	3606	–1.35	2.96E–04	7.10E–03	interleukin 18
220745_at	IL-19	29949	–1.34	2.75E–02	1.77E–01	interleukin 19
221111_at	IL-26	55801	–1.35	8.10E–03	8.11E–02	interleukin 26
203828_s_at	IL-32[¥]	9235	–1.45	4.17E–04	9.30E–03	interleukin 32
220322_at	IL-36G	56300	–1.38	3.96E–02	2.20E–01	interleukin 36, gamma
206975_at	LTA	4049	–1.29	7.47E–05	2.36E–03	lymphotoxin alpha
207339_s_at	LTB	4050	–1.38	2.93E–03	3.97E–02	lymphotoxin beta (TNF superfamily, member 3)
203085_s_at	TGFB1	7040	–1.15	1.40E–03	2.35E–02	transforming growth factor beta 1
207113_s_at	TNF[¥]	7124	–1.86	6.00E–06	2.91E–04	tumour necrosis factor
229814_at	TNFAIP8[¥]	25816	–1.24	1.54E–03	2.51E–02	tumour necrosis factor, alpha-induced protein 8
227420_at	TNFAIP8L1[¥]	126282	–1.29	5.81E–04	1.20E–02	tumour necrosis factor, alpha-induced protein 8-like 1
241819_at	TNFSF8[¥]	944	–1.46	4.08E–04	9.14E–03	tumour necrosis factor (ligand) superfamily, member 8
210643_at	TNFSF11	8600	1.47	2.40E–03	3.45E–02	tumour necrosis factor (ligand) superfamily, member 11
223502_s_at	TNFSF13B[¥]	10673	1.64	2.18E–12	7.91E–10	tumour necrosis factor (ligand) superfamily, member 13b
207907_at	TNFSF14	8740	–1.36	6.81E–03	7.19E–02	tumour necrosis factor (ligand) superfamily, member 14
229242_at	TNFSF15[¥]	9966	–1.74	1.47E–05	6.20E–04	tumour necrosis factor (ligand) superfamily, member 15

Probe set_ID	Gene Symbol	Entrez Gene	Fold change vs. placebo	p value	pFDR value	Gene Title
**Matrix metalloproteinases**						
204475_at	MMP1	4312	–1.56	1.16E–03	2.03E–02	matrix metallopeptidase 1
201069_at	MMP2[¥]	4313	–1.31	1.22E–02	1.07E–01	matrix metallopeptidase 2
204259_at	MMP7[¥]	4316	–1.58	4.11E–04	9.17E–03	matrix metallopeptidase 7
204580_at	MMP12[¥]	4321	–1.47	1.56E–06	9.36E–05	matrix metallopeptidase 12
217279_x_at	MMP14[¥]	4323	–1.15	4.52E–05	1.56E–03	matrix metallopeptidase 14 (membrane-inserted)
207289_at	MMP25	64386	1.34	1.45E–02	1.19E–01	matrix metallopeptidase 25

*Figure S4. Network connection between all pro-inflammatory cytokines and matrix-metalloproteinases differentially expressed (FDR<0.05 or p<0.05 and |FC|>1.3) by either dose treatment and the PDE4 isoforms inhibited by CHF6001. Each node represents all the proteins produced by a single, protein-coding gene locus, edges represent proteins that jointly contribute to a shared function, and the information inside the circle describes protein structure; a red line indicates the presence of fusion evidence; a green line, neighbourhood evidence; a blue line, co-occurrence evidence; a magenta line, experimental evidence; a yellow line, text mining evidence; a light blue line, database evidence; a black line, co-expression evidence; a purple line, protein homology evidence. Red nodes: 800 µg BID, blue nodes: 1600 µg BID. A green halo around the nodes: downregulation, a red halo: upregulation. *on the nodes: p<0.05 and |FC|>1.3 in at least one dose.*

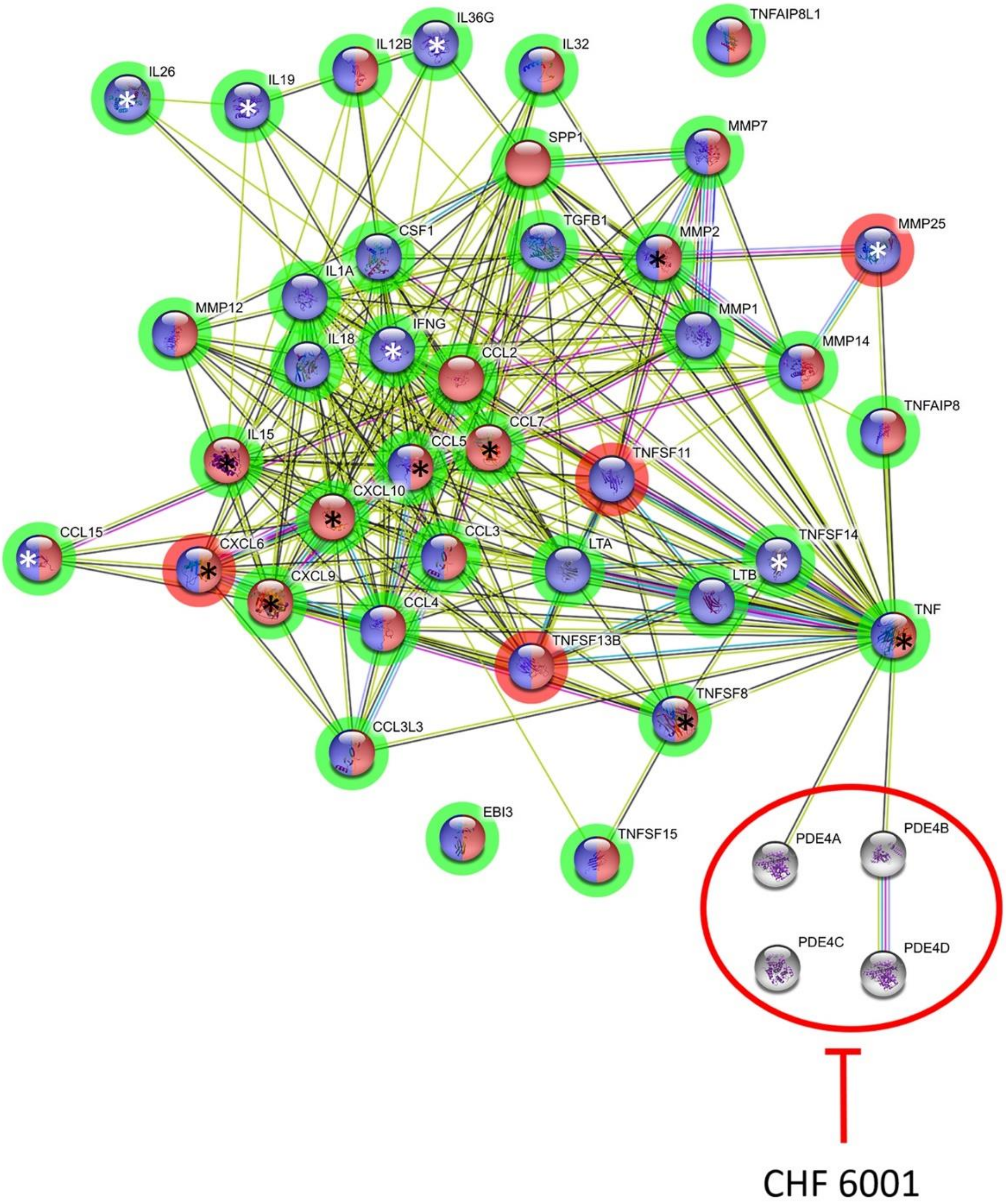

*Figure S5. Network of genes associated with the pathophysiology of COPD and differentially expressed (pFDR<0.05 or p<0.05 and |FC|>1.3) in favour of reduced inflammatory conditions after treatment with CHF6001 800 µg BID relative to placebo. Each node represents all the proteins produced by a single, protein-coding gene locus, edges represent proteins that jointly contribute to a shared function, and the information inside the circle describes protein structure. Edges: a red line indicates the presence of fusion evidence; a green line, neighbourhood evidence; a blue line, co-occurrence evidence; a magenta line, experimental evidence; a yellow line, text mining evidence; a light blue line, database evidence; a black line, co-expression evidence; a purple line, protein homology evidence. A green halo around the nodes: downregulation, a red halo: upregulation. *on the nodes: p<0.05 and |FC|>1.3*

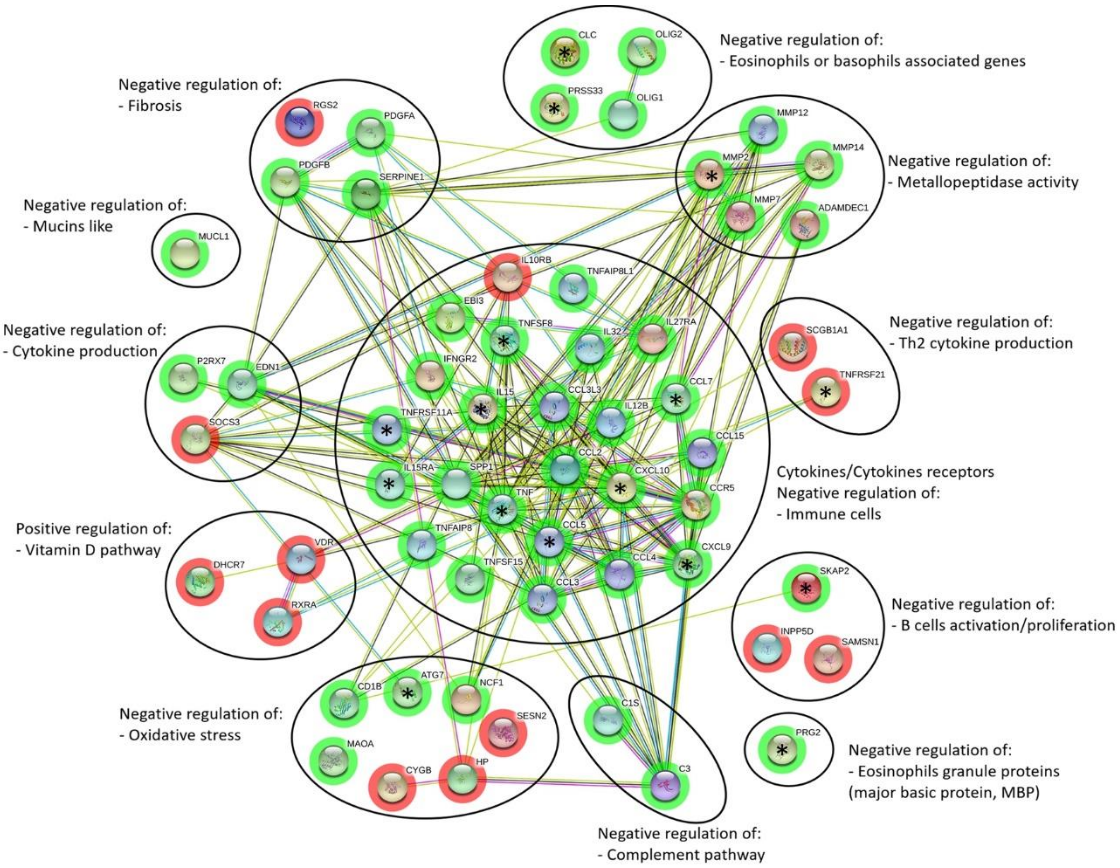

*Table S5. Genes associated with the pathophysiology of COPD and differentially expressed (pFDR<0.05 or p<0.05 and |FC|>1.3) in favour of reduced inflammatory conditions after treatment with CHF6001 800 µg BID relative to placebo. * common genes with CHF6001 1600 µg BID.*

Probe set_ID	Gene Symbol	Entrez Gene	Fold change vs. placebo	p value	pFDR value	Gene Title
**Negative regulation of eosinophil or basophil associated genes**						
206207_at	CLC*	1178	–1.33	4.90E–02	3.28E–01	Charcot-Leyden crystal galectin
228170_at	OLIG1*	116448	–2.16	7.38E–08	1.81E–05	oligodendrocyte transcription factor 1
213825_at	OLIG2*	10215	–2.02	7.42E–09	2.53E–06	oligodendrocyte lineage transcription factor 2
1552349_a_at	PRSS33*	260429	–1.34	1.37E–02	1.76E–01	protease, serine, 33
**Negative regulation of immune cells – Cytokines/Cytokines receptors**						
216598_s_at	CCL2	6347	–1.30	1.08E–03	3.70E–02	chemokine (C-C motif) ligand 2
205114_s_at	CCL3 /// CCL3L1 /// CCL3L3*	6348 /// 6349 /// 414062	–1.27	3.60E–05	2.89E–03	chemokine (C-C motif) ligand 3 /// chemokine (C-C motif) ligand 3-like 1 /// chemokine (C-C motif) ligand 3-like 3
204103_at	CCL4*	6351	–1.28	2.85E–05	2.38E–03	chemokine (C-C motif) ligand 4
204655_at	CCL5*	6352	–1.35	7.56E–03	1.27E–01	chemokine (C-C motif) ligand 5
208075_s_at	CCL7	6354	–1.32	2.56E–02	2.41E–01	chemokine (C-C motif) ligand 7

**Probe set_ID**	**Gene Symbol**	**Entrez Gene**	**Fold change vs. placebo**	**p value**	**pFDR value**	**Gene Title**
210390_s_at	CCL15 /// CCL15-CCL14*	6359 /// 348249	–1.81	3.78E–04	1.71E–02	chemokine (C-C motif) ligand 15 /// CCL15-CCL14 readthrough (NMD candidate)
206991_s_at	CCR5	1234	–1.29	5.78E–04	2.36E–02	chemokine (C-C motif) receptor 5 (gene/pseudogene)
203915_at	CXCL9	4283	–1.62	9.28E–03	1.43E–01	chemokine (C-X-C motif) ligand 9
204533_at	CXCL10	3627	–1.80	2.06E–02	2.16E–01	chemokine (C-X-C motif) ligand 10
219424_at	EBI3*	10148	–1.42	5.08E–05	3.82E–03	Epstein-Barr virus induced 3
201642_at	IFNGR2*	3460	–1.11	1.29E–03	4.20E–02	interferon gamma receptor 2 (interferon gamma transducer 1)
209575_at	IL-10RB*	3588	1.13	1.48E–03	4.60E–02	interleukin 10 receptor, beta
207901_at	IL-12B*	3593	–2.23	4.37E–06	5.09E–04	interleukin 12B
205992_s_at	IL-15	3600	–1.32	5.14E–03	1.01E–01	interleukin 15
207375_s_at	IL-15RA	3601	–1.41	9.41E–03	1.44E–01	interleukin 15 receptor, alpha
205926_at	IL-27RA*	9466	–1.30	2.21E–04	1.14E–02	interleukin 27 receptor, alpha
203828_s_at	IL-32*	9235	–1.46	4.22E–04	1.86E–02	interleukin 32
1568574_x_at	SPP1	6696	–1.27	6.89E–04	2.70E–02	secreted phosphoprotein 1
207113_s_at	TNF*	7124	–1.37	1.78E–02	2.01E–01	tumour necrosis factor

Probe set_ID	Gene Symbol	Entrez Gene	Fold change vs. placebo	p value	pFDR value	Gene Title
229814_at	TNFAIP8*	25816	–1.33	4.18E–05	3.23E–03	tumour necrosis factor, alpha-induced protein 8
227420_at	TNFAIP8L1*	126282	–1.35	8.20E–05	5.49E–03	tumour necrosis factor, alpha-induced protein 8-like 1
238846_at	TNFRSF11A	8792	–1.32	2.75E–02	2.50E–01	tumour necrosis factor receptor superfamily, member 11a, NFKB activator
241819_at	TNFSF8*	944	–1.33	6.75E–03	1.19E–01	tumour necrosis factor (ligand) superfamily, member 8
229242_at	TNFSF15*	9966	–1.62	1.79E–04	9.88E–03	tumour necrosis factor (ligand) superfamily, member 15
**Negative regulation of B cells activation/proliferation**						
1568943_at	INPP5D*	3635	1.73	1.65E–06	2.31E–04	inositol polyphosphate-5-phosphatase D
1569599_at	SAMSN1*	64092	1.44	1.30E–03	4.21E–02	SAM domain, SH3 domain and nuclear localisation signals 1
241331_at	SKAP2*	8935	–1.35	1.53E–02	1.86E–01	src kinase associated phosphoprotein 2
**Negative regulation of complement pathway**						
208747_s_at	C1S	716	–1.55	1.82E–04	9.99E–03	complement component 1, s subcomponent
217767_at	C3*	718	–1.28	3.43E–04	1.60E–02	complement component 3
**Negative regulation of cytokine production**						
222802_at	EDN1*	1906	–1.85	6.21E–05	4.42E–03	endothelin 1
207091_at	P2RX7*	5027	–1.27	3.19E–04	1.51E–02	purinergic receptor P2X, ligand gated ion channel, 7

Probe set_ID	Gene Symbol	Entrez Gene	Fold change vs. placebo	p value	pFDR value	Gene Title
206360_s_at	SOCS3*	9021	1.43	9.69E–04	3.41E–02	suppressor of cytokine signalling 3
**Negative regulation of eosinophils granule proteins**						
211743_s_at	PRG2*	5553	–1.36	6.27E–03	1.14E–01	proteoglycan 2, bone marrow (natural killer cell activator, eosinophil granule major basic protein)
**Negative regulation of fibrosis**						
205463_s_at	PDGFA*	5154	–1.32	1.22E–03	4.04E–02	platelet-derived growth factor alpha polypeptide
204200_s_at	PDGFB*	5155	–1.51	6.13E–09	2.18E–06	platelet-derived growth factor beta polypeptide
202388_at	RGS2*	5997	1.17	6.56E–08	1.63E–05	regulator of G-protein signalling 2
202627_s_at	SERPINE1*	5054	–1.32	2.98E–05	2.45E–03	serpin peptidase inhibitor, clade E (nexin, plasminogen activator inhibitor type 1), member 1
**Negative regulation of matrix metalloproteinases activity**						
206134_at	ADAMDEC1	27299	–1.53	7.08E–05	4.93E–03	ADAM-like, decysin 1
201069_at	MMP2*	4313	–1.32	1.28E–02	1.70E–01	Matrix peptidase 2
204259_at	MMP7*	4316	–1.71	5.44E–05	4.02E–03	Matrix peptidase 7
204580_at	MMP12*	4321	–1.36	1.05E–04	6.64E–03	Matrix peptidase 12
202827_s_at	MMP14*	4323	–1.30	6.09E–04	2.46E–02	Matrix peptidase 14 (membrane-inserted)

Probe set_ID	Gene Symbol	Entrez Gene	Fold change vs. placebo	p value	pFDR value	Gene Title
**Negative regulation of mucins like**						
1553602_at	MUCL1*	118430	–1.72	1.58E–05	1.47E–03	mucin-like 1
**Negative regulation of oxidative stress**						
1569827_at	ATG7*	10533	–1.34	1.05E–02	1.52E–01	autophagy related 7
206749_at	CD1B*	910	–1.56	1.03E–03	3.57E–02	CD1b molecule
1570410_at	CYGB*	114757	1.22	6.82E–04	2.69E–02	cytoglobin
206697_s_at	HP*	3240	1.74	4.18E–07	7.48E–05	haptoglobin
204388_s_at	MAOA	4128	–1.43	2.37E–04	1.20E–02	monoamine oxidase A
214084_x_at	NCF1*	653361	–1.36	2.40E–06	3.17E–04	neutrophil cytosolic factor 1
223195_s_at	SESN2*	83667	1.90	5.96E–15	3.78E–11	sestrin 2
**Negative regulation of Th2 cytokine production (IL-4, IL5, IL-13)**						
205725_at	SCGB1A1*	7356	1.75	1.09E–04	6.81E–03	secretoglobin, family 1A, member 1 (uteroglobin)
214581_x_at	TNFRSF21*	27242	1.32	3.92E–03	8.58E–02	tumour necrosis factor receptor superfamily, member 21
**Positive regulation of vitamin D pathway**						
201790_s_at	DHCR7*	1717	1.42	3.29E–06	4.07E–04	7-dehydrocholesterol reductase
202426_s_at	RXRA*	6256	1.30	2.28E–04	1.17E–02	retinoid X receptor alpha

Probe set_ID	Gene Symbol	Entrez Gene	Fold change vs. placebo	p value	pFDR value	Gene Title
213692_s_at	VDR*	7421	1.35	6.81E–06	7.37E–04	vitamin D (1,25- dihydroxyvitamin D3) receptor

Abbreviations: BID, twice daily.

*Figure S6. Network of genes associated with the pathophysiology of COPD, differentially expressed (pFDR<0.05 or p<0.05 and |FC|>1.3) in favour of reduced inflammatory conditions after treatment with CHF6001 1600 µg BID relative to placebo. Each node represents all the proteins produced by a single, protein-coding gene locus, edges represent proteins that jointly contribute to a shared function, and the information inside the circle describes protein structure. Edges: a red line indicates the presence of fusion evidence; a green line, neighbourhood evidence; a blue line, co-occurrence evidence; a magenta line, experimental evidence; a yellow line, text mining evidence; a light blue line, database evidence; a black line, co-expression evidence; a purple line, protein homology evidence. A green halo around the nodes: downregulation, a red halo: upregulation * on the nodes: p<0.05 and |FC|>1.3*

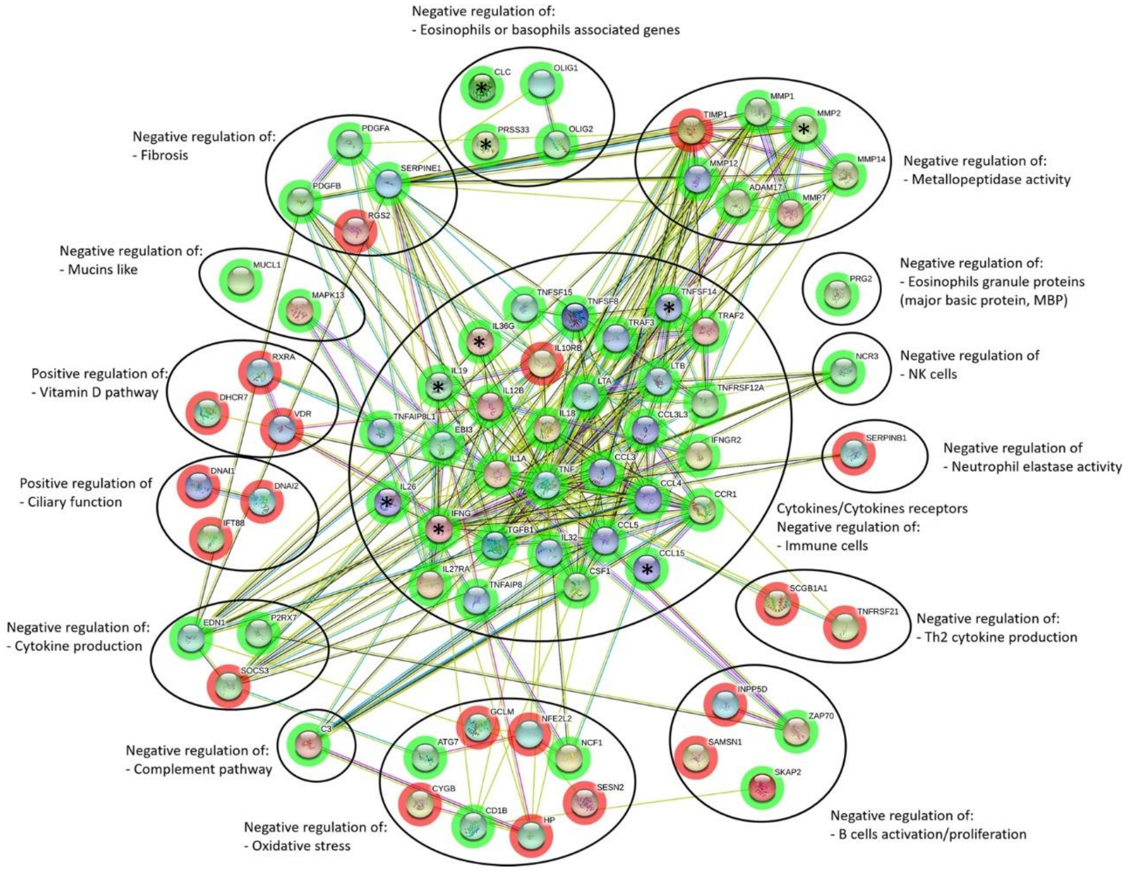

*Table S6. Genes associated with the pathophysiology of COPD and differentially expressed in favour of reduced inflammatory conditions after treatment with CHF6001 1600 µg BID relative to placebo. * common genes with CHF6001 800 µg BID.*

Probe set_ID	Gene Symbol	Entrez Gene	Fold change vs. placebo	p value	pFDR value	Gene Title
**Negative regulation of eosinophil or basophil related genes**						
206207_at	CLC*	1178	–1.36	2.93E–02	1.84E–01	Charcot-Leyden crystal galectin
228170_at	OLIG1*	116448	–2.25	1.37E–08	1.52E–06	oligodendrocyte transcription factor 1
213825_at	OLIG2*	10215	–2.39	4.50E–12	1.48E–09	oligodendrocyte lineage transcription factor 2
1552349_a_at	PRSS33*	260429	–1.32	1.70E–02	1.32E–01	protease, serine, 33
**Negative regulation of immune cells – Cytokines/Cytokines receptors**						
205114_s_at	CCL3 /// CCL3L1 /// CCL3L3*	6348 /// 6349 /// 414062	–1.39	3.67E–08	3.63E–06	chemokine (C-C motif) ligand 3 /// chemokine (C-C motif) ligand 3-like 1 /// chemokine (C-C motif) ligand 3-like 3
204103_at	CCL4*	6351	–1.38	1.63E–07	1.34E–05	chemokine (C-C motif) ligand 4
1555759_a_at	CCL5*	6352	–1.42	2.10E–03	3.13E–02	chemokine (C-C motif) ligand 5
210390_s_at	CCL15 /// CCL15-CCL14*	6359 /// 348249	–1.50	1.16E–02	1.03E–01	chemokine (C-C motif) ligand 15 /// CCL15-CCL14 readthrough (NMD candidate)
205099_s_at	CCR1	1230	–1.30	6.41E–05	2.07E–03	chemokine (C-C motif) receptor 1

Probe set_ID	Gene Symbol	Entrez Gene	Fold change vs. placebo	p value	pFDR value	Gene Title
209716_at	CSF1	1435	–1.35	1.53E–03	2.50E–02	colony stimulating factor 1 (macrophage)
219424_at	EBI3*	10148	–1.35	3.46E–04	8.00E–03	Epstein-Barr virus induced 3
210354_at	IFNG	3458	–1.52	1.85E–02	1.39E–01	interferon, gamma
201642_at	IFNGR2*	3460	–1.13	3.59E–04	8.25E–03	interferon gamma receptor 2 (interferon gamma transducer 1)
210118_s_at	IL-1A	3552	–1.53	1.51E–03	2.48E–02	interleukin 1 alpha
209575_at	IL-10RB*	3588	1.14	3.27E–04	7.66E–03	interleukin 10 receptor, beta
207901_at	IL-12B*	3593	–1.86	2.83E–04	6.84E–03	interleukin 12B
206295_at	IL-18	3606	–1.35	2.96E–04	7.10E–03	interleukin 18
220745_at	IL-19	29949	–1.34	2.75E–02	1.77E–01	interleukin 19
221111_at	IL-26	55801	–1.35	8.10E–03	8.11E–02	interleukin 26
205926_at	IL-27RA*	9466	–1.32	1.00E–04	2.98E–03	interleukin 27 receptor, alpha
203828_s_at	IL-32*	9235	–1.45	4.17E–04	9.30E–03	interleukin 32
220322_at	IL-36G	56300	–1.38	3.96E–02	2.20E–01	interleukin 36, gamma
206975_at	LTA	4049	–1.29	7.47E–05	2.36E–03	lymphotoxin alpha
207339_s_at	LTB	4050	–1.38	2.93E–03	3.97E–02	lymphotoxin beta (TNF superfamily, member 3)
203085_s_at	TGFB1	7040	–1.15	1.40E–03	2.35E–02	transforming growth factor beta 1

Probe set_ID	Gene Symbol	Entrez Gene	Fold change vs. placebo	p value	pFDR value	Gene Title
207113_s_at	TNF*	7124	–1.86	6.00E–06	2.91E–04	tumour necrosis factor
229814_at	TNFAIP8*	25816	–1.24	1.54E–03	2.51E–02	tumour necrosis factor, alpha-induced protein 8
227420_at	TNFAIP8L1*	126282	–1.29	5.81E–04	1.20E–02	tumour necrosis factor, alpha-induced protein 8-like 1
218368_s_at	TNFRSF12A	51330	–1.20	3.40E–04	7.89E–03	tumour necrosis factor receptor superfamily, member 12A
241819_at	TNFSF8*	944	–1.46	4.08E–04	9.14E–03	tumour necrosis factor (ligand) superfamily, member 8
207907_at	TNFSF14	8740	–1.36	6.81E–03	7.19E–02	tumour necrosis factor (ligand) superfamily, member 14
229242_at	TNFSF15*	9966	–1.74	1.47E–05	6.20E–04	tumour necrosis factor (ligand) superfamily, member 15
204413_at	TRAF2	7186	–1.11	9.45E–04	1.74E–02	TNF receptor-associated factor 2
208315_x_at	TRAF3	7187	–1.15	1.65E–03	2.64E–02	TNF receptor-associated factor 3
**Negative regulation of B cells activation/proliferation**						
1568943_at	INPP5D*	3635	2.16	1.25E–10	2.60E–08	inositol polyphosphate-5-phosphatase D
1555638_a_at	SAMSN1*	64092	1.38	1.93E–08	2.06E–06	SAM domain, SH3 domain and nuclear localisation signals 1
241331_at	SKAP2*	8935	–1.51	8.95E–04	1.67E–02	src kinase associated phosphoprotein 2
1555613_a_at	ZAP70	7535	–1.11	1.28E–03	2.20E–02	zeta chain of T cell receptor associated protein kinase 70kDa
**Negative regulation of complement pathway**						
217767_at	C3*	718	–1.29	1.78E–04	4.76E–03	complement component 3

Probe set_ID	Gene Symbol	Entrez Gene	Fold change vs. placebo	p value	pFDR value	Gene Title
**Negative regulation of cytokine production**						
222802_at	EDN1*	1906	–2.08	1.83E–06	1.07E–04	endothelin 1
207091_at	P2RX7*	5027	–1.35	5.97E–06	2.90E–04	purinergic receptor P2X, ligand gated ion channel, 7
206359_at	SOCS3*	9021	1.42	1.08E–05	4.77E–04	suppressor of cytokine signalling 3
**Negative regulation of eosinophils granule proteins**						
211743_s_at	PRG2*	5553	–1.38	3.38E–03	4.38E–02	proteoglycan 2, bone marrow (natural killer cell activator, eosinophil granule major basic protein)
**Negative regulation of fibrosis**						
205463_s_at	PDGFA*	5154	–1.33	8.77E–04	1.64E–02	platelet-derived growth factor alpha polypeptide
204200_s_at	PDGFB*	5155	–1.48	1.03E–08	1.19E–06	platelet-derived growth factor beta polypeptide
202388_at	RGS2*	5997	1.22	9.04E–11	2.02E–08	regulator of G-protein signalling 2
202628_s_at	SERPINE1*	5054	–1.44	6.94E–05	2.22E–03	serpin peptidase inhibitor, clade E (nexin, plasminogen activator inhibitor type 1), member 1
**Negative regulation of matrix metalloproteinases activity**						
213532_at	ADAM17	6868	–1.12	6.10E–04	1.25E–02	ADAM metallopeptidase domain 17
204475_at	MMP1	4312	–1.56	1.16E–03	2.03E–02	matrix metallopeptidase 1

Probe set_ID	Gene Symbol	Entrez Gene	Fold change vs. placebo	p value	pFDR value	Gene Title
201069_at	MMP2*	4313	–1.31	1.22E–02	1.07E–01	matrix metallopeptidase 2
204259_at	MMP7*	4316	–1.58	4.11E–04	9.17E–03	matrix metallopeptidase 7
204580_at	MMP12*	4321	–1.47	1.56E–06	9.36E–05	matrix metallopeptidase 12
217279_x_at	MMP14*	4323	–1.15	4.52E–05	1.56E–03	matrix metallopeptidase 14 (membrane-inserted)
201666_at	TIMP1	7076	1.12	2.49E–04	6.19E–03	TIMP metallopeptidase inhibitor 1
**Negative regulation of mucins like**						
210059_s_at	MAPK13	5603	–1.21	2.19E–05	8.62E–04	mitogen-activated protein kinase 13
1553602_at	MUCL1*	118430	–1.44	2.18E–03	3.22E–02	mucin-like 1
**Negative regulation of neutrophil elastase activity**						
239213_at	SERPINB1	1992	1.56	1.01E–04	3.01E–03	serpin peptidase inhibitor, clade B (ovalbumin), member 1
**Negative regulation of NK cells**						
211010_s_at	NCR3	259197	–1.14	2.67E–03	3.74E–02	natural cytotoxicity triggering receptor 3
**Negative regulation of oxidative stress**						
1569827_at	ATG7*	10533	–1.37	3.63E–03	4.62E–02	autophagy related 7
206749_at	CD1B*	910	–1.56	9.09E–04	1.69E–02	CD1b molecule
1570410_at	CYGB*	114757	1.20	1.77E–03	2.77E–02	cytoglobin

Probe set_ID	Gene Symbol	Entrez Gene	Fold change vs. placebo	p value	pFDR value	Gene Title
203925_at	GCLM	2730	1.52	5.88E–06	2.86E–04	glutamate-cysteine ligase, modifier subunit
206697_s_at	HP*	3240	1.39	1.06E–03	1.90E–02	haptoglobin
214084_x_at	NCF1*	653361	–1.35	3.70E–06	1.94E–04	neutrophil cytosolic factor 1
201146_at	NFE2L2	4780	1.15	1.89E–03	2.90E–02	nuclear factor, erythroid 2-like 2
223195_s_at	SESN2*	83667	2.22	3.69E–20	3.78E–16	sestrin 2
**Negative regulation of Th2 cytokines production (IL-4, IL5, IL-13)**						
205725_at	SCGB1A1*	7356	1.58	1.05E–03	1.88E–02	secretoglobin, family 1A, member 1 (uteroglobin)
214581_x_at	TNFRSF21*	27242	1.44	1.27E–04	3.61E–03	tumour necrosis factor receptor superfamily, member 21
**Positive regulation of ciliary function**						
220125_at	DNAI1	27019	1.50	1.13E–03	1.98E–02	dynein, axonemal, intermediate chain 1
220636_at	DNAI2	64446	1.49	1.89E–04	4.97E–03	dynein, axonemal, intermediate chain 2
204703_at	IFT88	8100	1.23	2.74E–04	6.69E–03	intraflagellar transport 88
**Positive regulation of vitamin D pathway**						
201790_s_at	DHCR7*	1717	1.50	6.66E–08	6.14E–06	7-dehydrocholesterol reductase
202426_s_at	RXRA*	6256	1.29	3.41E–04	7.91E–03	retinoid X receptor alpha
204254_s_at	VDR*	7421	1.30	6.28E–08	5.81E–06	vitamin D (1,25- dihydroxyvitamin D3) receptor

References

1. Irizarry RA, Bolstad BM, Collin F, Cope LM, Hobbs B, Speed TP. Summaries of Affymetrix GeneChip probe level data. Nucleic Acids Res. 2003;31:e15.

2. Stockley RA, Wiley InterScience (Online service). Chronic obstructive pulmonary disease. 2007.

3. Barnes PJ, Drazen JM, Rennard SI, Thomson NC, editors. Asthma and COPD: Basic mechanisms and clinical management. 2nd ed. [Oxford, UK]: Academic Press; 2008.

4. Sotgiu G, Radovanovic D, Santus P. Promise and limitations of relaxin-based therapies in chronic fibrotic lung diseases. Am. J. Respir. Crit. Care Med. 2007;20:258–64.

5. Sundar IK, Hwang J-W, Wu S, Sun J, Rahman I. Deletion of vitamin D receptor leads to premature emphysema/COPD by increased matrix metalloproteinases and lymphoid aggregates formation. Biochem. Biophys. Res. Commun. 2011;406:127–33. doi:10.1016/j.bbrc.2011.02.011

6. Ishii M, Yamaguchi Y, Nakamura T, Akishita M. The vitamin D receptors may function as antiinflammatory effects in patients with COPD. Chest 2015;148:690. doi:10.1378/chest.2249480

7. García de Tena J, El Hachem Debek A, Hernández Gutiérrez C, Izquierdo Alonso JL. The role of vitamin D in chronic obstructive pulmonary disease, asthma and other respiratory diseases. Arch. Bronconeumol. 2014;50:179–84. doi:10.1016/j.arbr.2014.03.015

8. Yew-Booth L, Birrell MA, Lau MS, Baker K, Jones V, Kilty I, et al. JAK-STAT pathway activation in COPD. Eur. Respir. J. 2015;46:843–5. doi:10.1183/09031936.00228414

9. Yanagisawa S, Baker JR, Vuppusetty C, Fenwick P, Donnelly LE, Ito K, et al. Decreased phosphatase PTEN amplifies PI3K signaling and enhances proinflammatory cytokine release in COPD. Am J Physiol Lung Cell Mol Physiol 2017;313:230–9. doi:10.1152/ajplung.00382.2016.-The

10. Walker JKL, Fisher JT. Editorial overview: Respiratory: GPCR signaling and the lung. Curr. Opin. Pharmacol. 2014;16:iv–vi. doi:10.1016/j.coph.2014.05.006

11. Oldenburger A, Roscioni SS, Jansen E, Menzen MH, Halayko AJ, Timens W, et al. Anti-inflammatory role of the cAMP effectors Epac and PKA: Implications in chronic obstructive pulmonary disease. PLoS One 2012;7:e31574. doi:10.1371/journal.pone.0031574

12. Brown WM. Treating COPD with PDE 4 inhibitors. Int. J. Chron. Obstruct. Pulmon. Dis. 2007;2:517–33.

13. Barnes PJ. The cytokine network in asthma and chronic obstructive pulmonary disease. J. Clin. Invest. 2008;118:3546–56. doi:10.1172/JCI36130

14. Belvisi MG, Mitchell JA. Targeting PPAR receptors in the airway for the treatment of inflammatory lung disease. Br. J. Pharmacol. 2009;158:994–1003. doi:10.1111/j.1476-5381.2009.00373.x

15. Siafakas N, editor. Management of Chronic Obstructive Pulmonary Disease. [Lausanne, Switzerland]: European Respiratory Society; 2006.

16. Medina-Tato DA, Ward SG, Watson ML. Phosphoinositide 3-kinase signalling in lung disease: leucocytes and beyond. Immunology 2007;121:448–61. doi:10.1111/j.1365-2567.2007.02663.x

17. Murray PJ. The JAK-STAT signaling pathway: Input and output integration. J. Immunol. 2007;178:2623–9. doi:10.4049/JIMMUNOL.178.5.2623

18. Barnes PJ. Inflammatory mechanisms in patients with chronic obstructive pulmonary

disease. J. Allergy Clin. Immunol. 2016;138:16–27. doi:10.1016/j.jaci.2016.05.011

19. Costa C, Traves SL, Tudhope SJ, Fenwick PS, Belchamber KBR, Russell REK, et al. Enhanced monocyte migration to CXCR3 and CCR5 chemokines in COPD. Eur. Respir. J. 2016;47:1093–102. doi:10.1183/13993003.01642-2015

20. Radsak MP, Taube C, Haselmayer P, Tenzer S, Salih HR, Wiewrodt R, et al. Soluble triggering receptor expressed on myeloid cells 1 is released in patients with stable chronic obstructive pulmonary disease. Clin. Dev. Immunol. 2007;2007:52040. doi:10.1155/2007/52040

21. Freeman CM, Martinez FJ, Han MK, Ames TM, Chensue SW, Todt JC, et al. Lung dendritic cell expression of maturation molecules increases with worsening chronic obstructive pulmonary disease. Am. J. Respir. Crit. Care Med. 2009;180:1179–88. doi:10.1164/rccm.200904-0552OC

22. Kanazawa H, Tochino Y, Asai K, Hirata K. Simultaneous assessment of hepatocyte growth factor and vascular endothelial growth factor in epithelial lining fluid from patients with COPD. Chest 2014;146:1159–65. doi:10.1378/chest.14-0373

23. Faner R, Sobradillo P, Noguera A, Gomez C, Cruz T, López-Giraldo A, et al. The inflammasome pathway in stable COPD and acute exacerbations. ERJ Open Research 2016;2:00002–2016. doi:10.1183/23120541.00002-2016

24. Pouwels SD, van Geffen WH, Jonker MR, Kerstjens HAM, Nawijn MC, Heijink IH. Increased neutrophil expression of pattern recognition receptors during COPD exacerbations. Respirology 2017;22:401–4. doi:10.1111/resp.12912

25. Kinose D, Ogawa E, Kudo M, Marumo S, Kiyokawa H, Hoshino Y, et al. Association of COPD exacerbation frequency with gene expression of pattern recognition receptors in inflammatory cells in induced sputum. Clin. Respir. J. 2016;10:11–21. doi:10.1111/crj.12171

26. Culley FJ. Natural killer cells in infection and inflammation of the lung. Immunology 2009;128:151–63. doi:10.1111/j.1365-2567.2009.03167.x

27. Kim JH, Jang YJ. Role of natural killer cells in airway inflammation. Allergy. Asthma Immunol. Res. 2018;10:448–56. doi:10.4168/aair.2018.10.5.448

28. Carman C V. Mechanisms for transcellular diapedesis: probing and pathfinding by `invadosome-like protrusions'. J. Cell Sci. 2009;122:3025–35. doi:10.1242/jcs.047522

29. Gangemi S, Casciaro M, Trapani G, Quartuccio S, Navarra M, Pioggia G, et al. Association between HMGB1 and COPD: A Systematic Review. Mediators Inflamm. 2015;2015:164913. doi:10.1155/2015/164913

30. Rahman I, MacNee W. Role of transcription factors in inflammatory lung diseases. Thorax 1998;53:601–12. doi:10.1136/THX.53.7.601

31. Schuliga M. NF-kappaB signaling in chronic inflammatory airway disease. Biomolecules 2015;5:1266–83. doi:10.3390/biom5031266

32. Domej W, Oettl K, Renner W. Oxidative stress and free radicals in COPD--implications and relevance for treatment. Int. J. Chron. Obstruct. Pulmon. Dis. 2014;9:1207–24. doi:10.2147/COPD.S51226

33. Chen J, Liao M, Gao X, Zhong Q, Tang T, Yu X, et al. IL-17A induces pro-inflammatory cytokines production in macrophages via MAPKinases, NF-κB and AP-1. Cell. Physiol. Biochem. 2013;32:1265–74. doi:10.1159/000354525

34. Eustace A, Smyth LJC, Mitchell L, Williamson K, Plumb J, Singh D. Identification of cells expressing IL-17A and IL-17F in the lungs of patients with COPD. Chest 2011;139:1089–100. doi:10.1378/CHEST.10-0779

35. Nixon J, Newbold P, Mustelin T, Anderson GP, Kolbeck R. Monoclonal antibody therapy

for the treatment of asthma and chronic obstructive pulmonary disease with eosinophilic inflammation. Pharmacol. Ther. 2017;169:57–77. doi:10.1016/J.PHARMTHERA.2016.10.016

36. Pinto-Plata V, Toso J, Lee K, Park D, Bilello J, Mullerova H, et al. Profiling serum biomarkers in patients with COPD: associations with clinical parameters. Thorax 2007;62:595–601. doi:10.1136/thx.2006.064428

37. Mercer BA, D'Armiento JM. Emerging role of MAP kinase pathways as therapeutic targets in COPD. Int. J. Chron. Obstruct. Pulmon. Dis. 2006;1:137–50.

38. Fawzy A, Putcha N, Paulin LM, Aaron CP, Labaki WW, Han MK, et al. Association of thrombocytosis with COPD morbidity: the SPIROMICS and COPDGene cohorts. Respir. Res. 2018;19:20. doi:10.1186/s12931-018-0717-z

39. Karagiannis K, Proklou A, Tsitoura E, Lasithiotaki I, Kalpadaki C, Moraitaki D, et al. Impaired mRNA expression of the migration related chemokine receptor cxcr4 in mesenchymal stem cells of COPD patients. Int. J. Inflam. 2017;2017:1–8. doi:10.1155/2017/6089425

40. Jubrail J, Kurian N, Niedergang F. Macrophage phagocytosis cracking the defect code in COPD. Biomed. J. 2017;40:305–12. doi:10.1016/j.bj.2017.09.004

41. Yoshikawa T, Dent G, Ward J, Angco G, Nong G, Nomura N, et al. Impaired neutrophil chemotaxis in chronic obstructive pulmonary disease. Am. J. Respir. Crit. Care Med. 2007;175:473–9. doi:10.1164/rccm.200507-1152OC

42. Markoulaki D, Kostikas K, Papatheodorou G, Koutsokera A, Alchanatis M, Bakakos P, et al. Hemoglobin, erythropoietin and systemic inflammation in exacerbations of chronic obstructive pulmonary disease. Eur. J. Intern. Med. 2011;22:103–7. doi:10.1016/j.ejim.2010.07.010

43. Shimbara A, Christodoulopoulos P, Soussi-Gounni A, Olivenstein R, Nakamura Y, Levitt RC, et al. IL-9 and its receptor in allergic and nonallergic lung disease: Increased expression in asthma. J. Allergy Clin. Immunol. 2000;105:108–15. doi:10.1016/S0091-6749(00)90185-4

44. O'Donnell RA, Richter A, Ward J, Angco G, Mehta A, Rousseau K, et al. Expression of ErbB receptors and mucins in the airways of long term current smokers. Thorax 2004;59:1032–40. doi:10.1136/thx.2004.028043

45. Creutzberg EC, Casaburi R. Endocrinological disturbances in chronic obstructive pulmonary disease. Eur. Respir. J. Suppl. 2003;46:76s-80s. doi:10.1183/09031936.03.00004610

46. Holden NS, George T, Rider CF, Chandrasekhar A, Shah S, Kaur M, et al. Induction of regulator of G-Protein Signaling 2 expression by long-acting 2-adrenoceptor agonists and glucocorticoids in human airway epithelial cells. J. Pharmacol. Exp. Ther. 2013;348:12–24. doi:10.1124/jpet.113.204586

47. Pomerenke A, Lea SR, Herrick S, Lindsay MA, Singh D. Characterization of TLR-induced inflammatory responses in COPD and control lung tissue explants. Int. J. Chron. Obstruct. Pulmon. Dis. 2016;11:2409–17. doi:10.2147/COPD.S105156

48. Shaykhiev R, Salit J, Crystal RG. Small airway epithelium of smokers with COPD exhibits an altered cell cycle−related gene expression profile. Am. J. Respir. Crit. Care Med. 2009;179:A1905.

49. Chand S, Mehta N, Bahia MS, Dixit A, Silakari O. Protein kinase C-theta inhibitors: a novel therapy for inflammatory disorders. Curr. Pharm. Des. 2012;18:4725–46.

50. Biljak VR, Rumora L, Čepelak I, Pancirov D, Popović-Grle S, Sorić J, et al. Glutathione cycle in stable chronic obstructive pulmonary disease. Cell Biochem. Funct. 2010;28:448–53. doi:10.1002/cbf.1675

51. Petrusca DN, Gu Y, Adamowicz JJ, Rush NI, Hubbard WC, Smith PA, et al. Sphingolipid-mediated inhibition of apoptotic cell clearance by alveolar macrophages. J. Biol. Chem. 2010;285:40322–32. doi:10.1074/jbc.M110.137604

52. Nurwidya F, Damayanti T, Yunus F. The role of innate and adaptive immune cells in the immunopathogenesis of chronic obstructive pulmonary disease. Tuberc. Respir. Dis. (Seoul). 2016;79:5–13. doi:10.4046/trd.2016.79.1.5

53. Stolarczyk M, Scholte BJ. The EGFR-ADAM17 axis in chronic obstructive pulmonary disease and cystic fibrosis lung pathology. Mediators Inflamm. 2018;2018:1–22. doi:10.1155/2018/1067134

54. Hodge S, Hodge G, Holmes M, Reynolds PN. Increased airway epithelial and T-cell apoptosis in COPD remains despite smoking cessation. Eur. Respir. J. 2005;25:447–54. doi:10.1183/09031936.05.00077604
